# Activation of mTORC1 in subchondral bone preosteoblasts promotes osteoarthritis by stimulating bone sclerosis and secretion of CXCL12

**DOI:** 10.1038/s41413-018-0041-8

**Published:** 2019-02-20

**Authors:** Chuangxin Lin, Liangliang Liu, Chun Zeng, Zhong-Kai Cui, Yuhui Chen, Pinling Lai, Hong Wang, Yan Shao, Haiyan Zhang, Rongkai Zhang, Chang Zhao, Hang Fang, Daozhang Cai, Xiaochun Bai

**Affiliations:** 1 Department of Orthopedics, Academy of Orthopedics-Guangdong Province, The Third Affiliated Hospital of Southern Medical University, 510630 Guangzhou, China; 2 Key Laboratory of Mental Health of the Ministry of Education, Department of Cell Biology, School of Basic Medical Sciences, Southern Medical University, 510515 Guangzhou, China; 3 Department of Orthopedic Surgery, Shantou Central Hospital, Affiliated Shantou Hospital of Sun Yat-Sen University, 515041 Shantou, China

**Keywords:** Pathogenesis, Bone

## Abstract

Increasing evidences show that aberrant subchondral bone remodeling plays an important role in the development of osteoarthritis (OA). However, how subchondral bone formation is activated and the mechanism by which increased subchondral bone turnover promotes cartilage degeneration during OA remains unclear. Here, we show that the mechanistic target of rapamycin complex 1 (mTORC1) pathway is activated in subchondral bone preosteoblasts (Osterix+) from OA patients and mice. Constitutive activation of mTORC1 in preosteoblasts by deletion of the mTORC1 upstream inhibitor, tuberous sclerosis 1, induced aberrant subchondral bone formation, and sclerosis with little-to-no effects on articular cartilage integrity, but accelerated post-traumatic OA development in mice. In contrast, inhibition of mTORC1 in preosteoblasts by disruption of Raptor (mTORC1-specific component) reduced subchondral bone formation and cartilage degeneration, and attenuated post-traumatic OA in mice. Mechanistically, mTORC1 activation promoted preosteoblast expansion and Cxcl12 secretion, which induced subchondral bone remodeling and cartilage degeneration during OA. A Cxcl12-neutralizing antibody reduced cartilage degeneration and alleviated OA in mice. Altogether, these findings demonstrate that mTORC1 activation in subchondral preosteoblasts is not sufficient to induce OA, but can induce aberrant subchondral bone formation and secrete of Cxcl12 to accelerate disease progression following surgical destabilization of the joint. Pharmaceutical inhibition of the pathway presents a promising therapeutic approach for OA treatment.

## Arthritis: Activation of mTORC1 promotes osteoarthritis by stimulating bone sclerosis

Mammalian target of rapamycin complex 1 (mTORC1) is a protein complex that controls protein synthesis, and activation of mTORC1 in subchondral preosteoblasts promotes arthritis by stimulating bone sclerosis (abnormal hardening) and secreting C-X-C motif chemokine 12 (CXCL12). Subchondral preosteoblasts are cells that differentiate into bone-forming cells below the cartilage in joints, and CXCL12 is a protein that regulates a wide range of cellular activities. A team headed by Daozhang Cai and Xiaochun Bai of The Third Affiliated Hospital of Southern Medical University, Guangzhou, China investigated mTORC1 activity in subchondral preosteoblasts from osteoarthritis patients and mice. The team found that mTORC1 activation of subchondral preosteoblasts promoted osteoarthritis by stimulating abnormal subchondral bone formation and secretion of CXCL12, promoting cartilage degeneration. The authors believe that pharmaceutical inhibition of that pathway offers a promising approach in treating osteoarthritis.

## Introduction

Osteoarthritis (OA) is a highly prevalent and degenerative joint disorder which mainly affects the weight-bearing joints such as hips and knees, and is the leading cause of physical disability,^1–3^ presenting an enormous clinical and financial burden. However, no effective disease-modifying treatment for OA is currently available until the disease reaches the end stage, necessitating joint replacement.^4^ Indeed, the development of OA disease affects all joint tissues, being characterized by progressive degeneration of articular cartilage, vascular invasion of the articular surface, subchondral bone remodeling, osteophyte formation, and synovial inflammation.^5^ Despite the identified risk factors, which include genetic predisposition,^6^ mechanical abnormality,^7^ age,^8^ and obesity,^9^ the exact pathogenesis of OA remains unclear.

Recently, increasing evidences indicate that articular cartilage degeneration is related to aberrant subchondral bone turnover.^1,10,11^ Subchondral bone provides mechanical support for the overlying articular cartilage during the movement of joints and absorbs most of the mechanical force transmitted by diarthrodial joints. Relative to the slower turnover rate of articular cartilage, subchondral bone undergoes more rapid modeling and remodeling in response to changes of the mechanical environment.^12^ Indeed, homeostasis and integrity of articular cartilage rely on its biochemical and biomechanical interplay with subchondral bone and other joint tissues,^13^ and treatment targeting articular cartilage alone in OA has proven to be insufficient to halt disease progression. In a situation of instability, such as occurring with ligament injury,^14^ excessive body weight,^15^ or weakening muscles related to aging,^16^ the mechanical loading on weight-bearing joints is dramatically increased, and changes in the subchondral bone microarchitecture may precede articular cartilage damage. Thus, changes in the subchondral bone have been suggested to predict the severity of cartilage damage in OA.^17^ However, the mechanism by which aberrant subchondral bone formation is induced and the interplay between increased subchondral bone turnover and articular cartilage degeneration during OA remains unclear.

Mechanistic target of rapamycin (mTOR) is a highly conserved Ser/Thr protein kinase that functions as a master regulator of cell growth, proliferation, survival, and metabolism in response to nutrients, growth factors, and stresses.^18,19^ mTOR forms two distinct functional complexes, mTOR complex 1 (mTORC1) and mTORC2. mTORC1 is a sensitive target of rapamycin and is suppressed by the functional complex, tuberous sclerosis complex 1/2 (TSC1/2).^20^ Accumulating evidences suggest that mTORC1 is essential for maintenance of chondrocyte metabolic homeostasis and its activation in articular chondrocyte plays a vital role in OA development.^21^ We have previously shown that mTORC1 coordinates chondrocyte growth, proliferation, and differentiation, and that mTORC1 activation in turn activates articular cartilage chondrocytes and initiates OA, while its deletion or inhibition in chondrocyte prevents OA development.^22,23^ Previous studies by our group and others have also shown that balanced mTORC1 activity is critical for bone metabolism and development, acting by regulating osteoblast and osteoclast proliferation, differentiation, and function.^24–26^ However, the role of mTORC1 in subchondral bone formation during the pathogenesis of OA and the underlying mechanisms have not been reported.

C–X–C motif chemokine 12 (Cxcl12), also known as the stromal cell-derived factor 1 (SDF1), is an 8-kDa chemokine originally isolated from bone marrow stromal cells.^27^ It regulates a wide variety of cellular activities via interactions with C–X–C chemokine receptor type 4 (CXCR4).^28^ Recently, increasing evidences indicate that Cxcl12/CXCR4 may play a role in the progression of OA. A dramatic increase of Cxcl12 is found in the synovial fluid in the knee joints of rheumatoid arthritis and OA patients.^29^ Secondly, in vitro experiments have demonstrated that Cxcl12 regulates chondrocyte catabolic activity by stimulating the release of matrix metalloproteinase-3 (MMP-3) and MMP-13.^29,30^ These findings strongly suggest that Cxcl12 induces articular cartilage matrix degeneration during OA development. However, the mechanism by which Cxcl12 is regulated in OA remains to be elucidated.

In this study, we demonstrate that mTORC1 is activated in the preosteoblasts of subchondral bone from OA patients and destabilized OA mice. mTORC1 activation in Osterix^+^ cells induces preosteoblasts to form aberrant clusters resulting in osteogenesis in subchondral bone, while inhibition of mTORC1 attenuates these pathological changes and reduces the degeneration of articular cartilage. mTORC1 activation of subchondral preosteoblasts promotes OA by stimulating aberrant subchondral bone formation and secretion of Cxcl12 to promote cartilage degeneration; thus, pharmaceutical inhibition of the pathway might be a promising therapeutic approach for OA treatment.

## Results

### Preosteoblasts accumulate in subchondral bone during OA development

To investigate the role of subchondral bone changes in OA, articular cartilage and subchondral bone specimens from OA patients undergoing total knee replacement surgery or from non-OA trauma patients were analyzed. Hematoxylin and eosin (H&E) and Safranin O-Fast green staining revealed aberrant bone formation and severe bone sclerosis in the tibial subchondral bone of OA patients (Supplementary Fig. 1a–c), which were confirmed by micro-computed tomography three-dimensional (3D) reconstruction and analysis (Supplementary Fig. 1h and i). Tibial subchondral bone volume/total volume (BV/TV) fraction was positively correlated with OA Research Society International (OARSI) scores (*r* = 0.863, *P* < 0.001) (Supplementary Fig. 1j). Importantly, the number of Osterix^+^ cells was dramatically increased in OA patients compared with that of non-OA control (Supplementary Fig. 1d–e), while the number of subchondral osteoclasts remained unchanged (Supplementary Fig. 1f–g), indicating that uncoupled subchondral bone modeling occurred during OA progression.

**Fig. 1:**
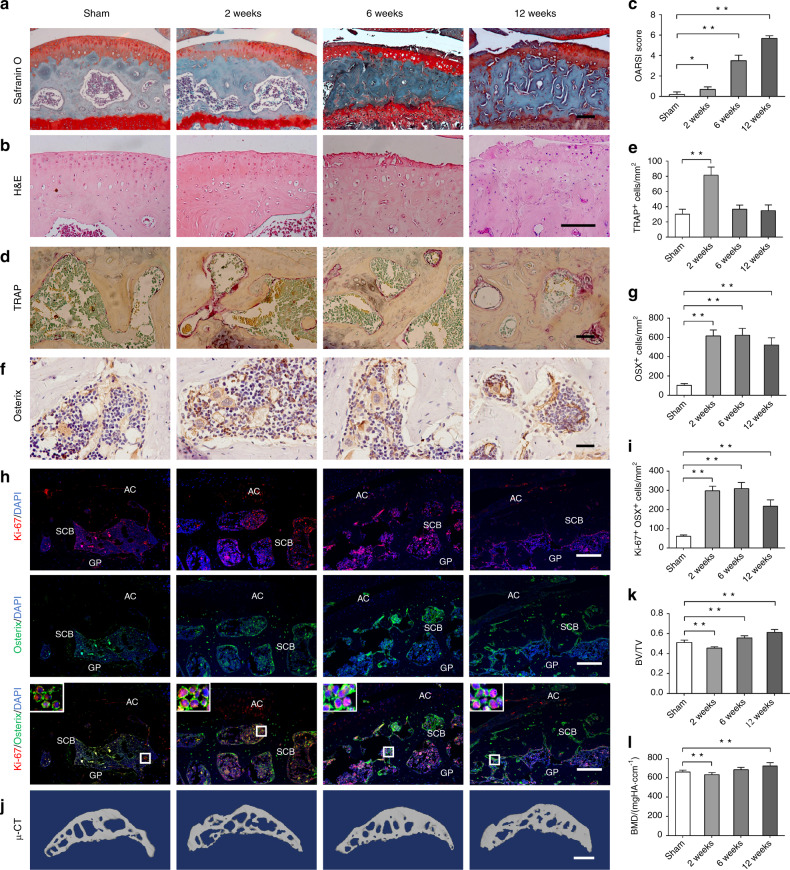
Preosteoblasts accumulate and activate in subchondral bone of ACLT OA mice. **a**, -**b** Representative Safranin O-Fast green and H&E staining of sagittal sections of tibia articular cartilage and subchondral bone in ACLT OA mice. Scale bars, 100 μm. **c** OARSI scores at 2, 6, and 12 weeks post surgery compared with that of the sham control. **d,** **e** TRAP staining and quantitative analysis of osteoclasts in mouse tibial subchondral bone at 2, 6, and 12 weeks post ACLT surgery compared with that of the sham control. Scale bars, 50 μm. **f**, **g** Representative immunohistochemical images and quantitative analysis of Osterix^+^ preosteoblasts in mouse tibial subchondral bone at 2, 6, and 12 weeks post ACLT surgery compared with that of the sham control. Scale bars, 100 μm. **h**, **i** Immunostaining images and quantitative analysis of Ki-67 in Osterix^+^ preosteoblasts in mouse tibial subchondral bone at 2, 6, and 12 weeks post ACLT surgery compared with that of the sham control. Boxed area is magnified on the top left corner. Scale bars, 100 μm. AC, articular cartilage; SCB, subchondral bone; GP, growth plate. **j** Representative 3D reconstructed micro-CT images of sagittal sections of mouse tibia subchondral bone at 2, 6, and 12 weeks post ACLT surgery compared with that of the sham control. Scale bars, 1mm. **k**, **l** Quantitative analysis of bone mass in subchondral bone: bone volume/total volume (BV/TV) and bone mineral density (BMD). Data are shown as mean±s.d. and analyzed by one-way ANOVA with Bonferroni post hoc test. *n* = 8, **P < 0.01

To further evaluate subchondral bone changes during OA progression, the anterior cruciate ligament (ACL) of mice was transected to generate a destabilized ACL-transected (ACLT) OA mouse model. Proteoglycan loss and articular cartilage degeneration were detected at 2 weeks after ACLT and progressed gradually (Fig. 1a–c). Interestingly, enhanced subchondral bone resorption in the tibial subchondral bone was observed at 2 weeks after ACLT, but aberrant bone formation and severe sclerosis occurred at the late stages of OA (Fig. 1a), as manifested by an increase in osteoclast number as well as larger subchondral bone marrow cavities at 2 weeks, and subsequently a decrease in the osteoclast number to baseline at 6 weeks after surgery (Fig. 1d, e). The number of Osterix^+^ cells, however, was continuously elevated from 2 to 12 weeks after surgery (Fig. 1f, g), which may result from enhanced proliferation of Osterix^+^ cells (Fig. 1h, i). Immunofluorescent analysis of Ki-67 in cultured primary preosteoblasts and mouse articular cartilage was used to rule out the false-positive results (Supplementary Fig. 2a). Micro-CT scan and analysis further confirmed that the BV/TV of tibial subchondral bone had decreased at 2 weeks, but was increased both at 6 and 12 weeks post surgery (Fig. 1j, k). Similarly, bone mineral density (BMD) was decreased at 2 weeks, but increased at 12 weeks post surgery (Fig. 1l). These findings further indicate that subchondral bone preosteoblasts are activated during OA, resulting in aberrant bone formation and sclerosis.

### mTORC1 is activated in subchondral bone preosteoblasts from OA patients and destabilized OA mice

**Fig. 2:**
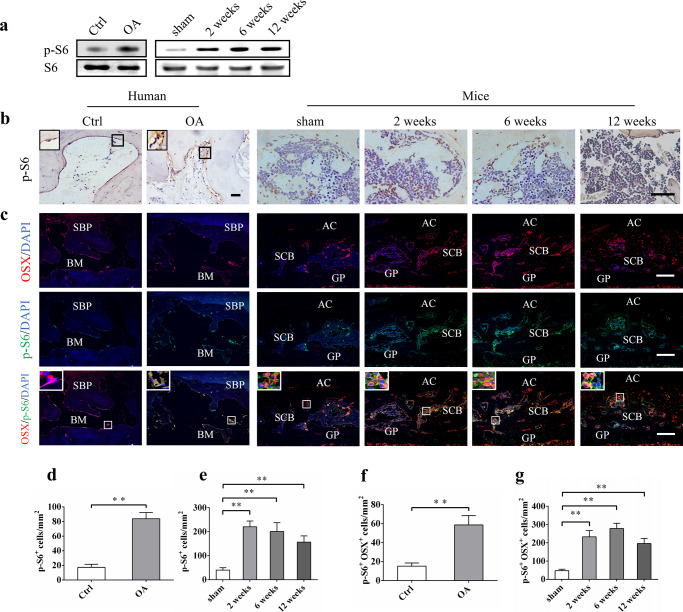
mTORC1 is activated in subchondral bone preosteoblasts in OA patients and destabilized OA mice. **a** Western blot analysis of p-S6 expression in tibia subchondral bone tissues from patients and OA mice. **b**, **d**, **e**) Representative immunostaining and quantitative analysis of p-S6^+^ cells in tibial subchondral bone of OA patients and OA mice. Scale bars, 200 μm. **c**, **f**, **g** Representative immunostaining and quantitative analysis of p-S6 in Osterix^+^ preosteoblasts in tibial subchondral bone of OA patients and OA mice. Boxed area is magnified on the top left corner. Scale bars, 100 μm. SBP, subchondral bone plate; BM, bone marrow; AC, articular cartilage; SCB, subchondral bone; GP, growth plate. Data are shown as mean±s.d. and analyze by Student’s *t* test or one-way ANOVA . *n* ≥8, **P < 0.01

Recent studies have shown that expression of mTOR is increased in peripheral blood mononuclear cells of OA patients, and is related to disease activity,^31^ and that pharmacological inhibition or genetic deletion of mTOR in chondrocyte decreases the severity of OA in an animal model.^16,32^ However, whether or not mTORC1 is activated in subchondral bone, and its role in OA has not been reported. Western blot analysis of subchondral bone tissues from collected tibial plateau specimens showed an enhanced phosphorylation of S6 (S235/236) (p-S6, a target and marker of mTORC1 activation) in OA patients compared to that of control (Fig. 2a). Consistently, immunohistochemical staining confirmed that the number of p-S6-positive cells dramatically increased in the subchondral bone of OA patients (Fig. 2b, d).

Activation of mTORC1 was also detected in tibial subchondral bone tissues isolated from ACLT mice^33^ at 2, 6, and 12 weeks post surgery (Fig. 2a). P-S6 staining revealed a dramatic increase of p-S6-positive cells as early as 2 weeks post surgery (Fig. 2b, e). Moreover, most p-S6-positive cells were located on the subchondral bone surface in both OA patients and mice. Osterix and p-S6 double staining confirmed that p-S6 was detected mainly in Osterix^+^ preosteoblasts in the subchondral bone of OA patients and OA mice (Fig. 2c, f, g). In addition, mTORC1 activation could also be detected in some mature osteoblasts (OCN^+^) during OA development in ACLT mice (Supplementary Fig. 2b–c). Taken together, these results indicate that mTORC1 is activated in subchondral bone, specifically in osteoblastic lineage cells in OA patients and mice.

### Constitutive activation of mTORC1 in preosteoblasts induces aberrant bone formation in subchondral bone and promotes OA in mice

**Fig. 3:**
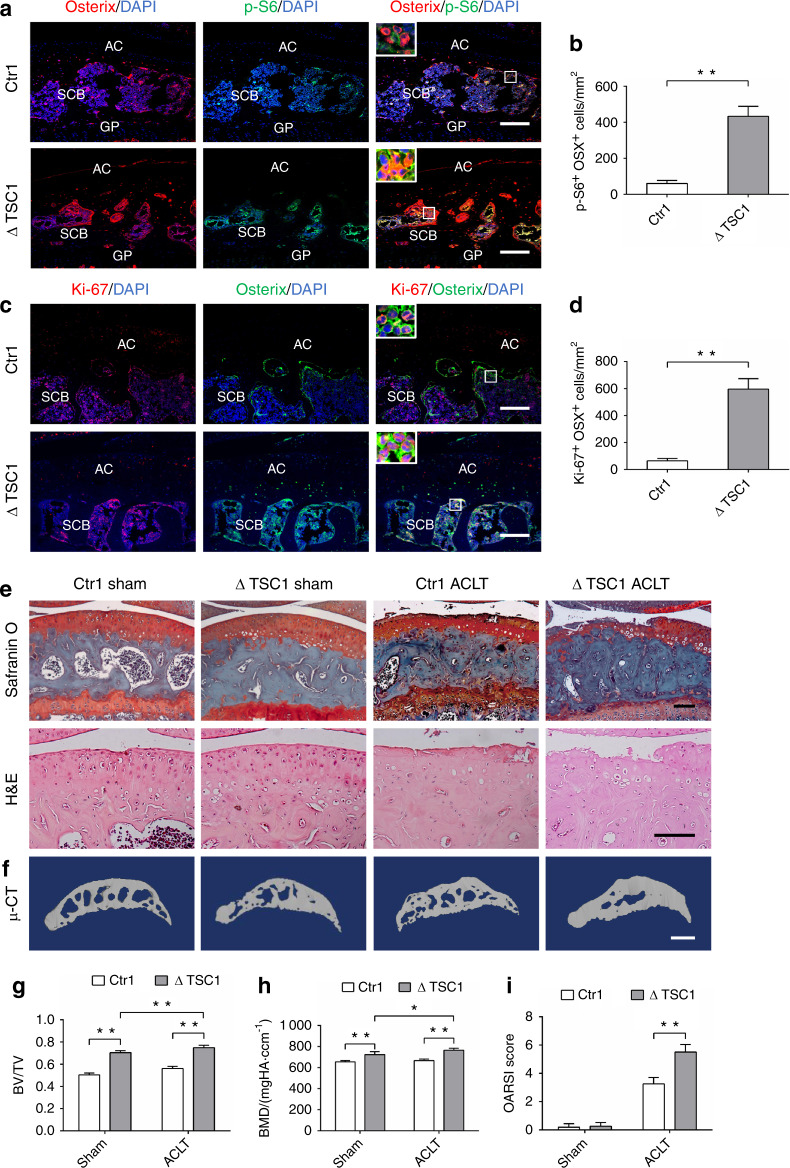
Constitutive activation of mTORC1 in preosteoblasts induces aberrant bone formation in subchondral bone and promotes OA in mice. **a**, **b** Representative photomicrographs and quantification of immunostaining of p-S6 in Osterix^+^ preosteoblasts in tibial subchondral bone of transgenic mice after Tsc1 deletion (ΔTSC1) compared to that of their littermates (Ctrl). **c**, **d** Representative photomicrographs and quantification of immunostaining of Ki-67 in Osterix^+^ preosteoblasts in tibial subchondral bone of transgenic mice after Tsc1 deletion (ΔTSC1) compared to that of their littermates (Ctrl). Boxed area is magnified on the top corner. Scale bars, 100μm. AC, articular cartilage; SCB, subchondral bone; GP, growth plate. **e** Representative Safranin O-Fast green and H&E staining of sagittal sections of articular cartilage and tibia subchondral bone of ΔTSC1 sham mice and ΔTSC1 ACLT mice compared to that of their littermates (Ctrl). Scale bars, 100 μm. **f** Representative 3D reconstructed micro-CT images of sagittal of tibia subchondral bone of ΔTSC1 sham mice and ΔTSC1 ACLT mice compared to that of their littermates (Ctrl). Scale bars, 1mm. **g**, **h** Quantitative analysis of bone mass in subchondral bone: bone volume/total volume (BV/TV) and bone mineral density (BMD). **i** OARSI scores based on the histology analysis. Data are shown as mean ± s.d. and analyzed by Student’s *t* test or two-way ANOVA . *n* ≥ 6, *P < 0.05, and **P < 0.01

To characterize the specific role of subchondral bone mTORC1 activation in OA development, we generated mice with inducible mTORC1 activation in preosteoblast cells committed to the osteoblast lineage. We crossed floxed TSC1 (mTORC1-negative regulator) mice with Osx1-GFP::Cre mice (which express a GFP-Cre fusion protein under the direction of the Osx promoter and doxycycline) to generate conditional TSC1-knockout mice. Male mice with the genotype Osx1-GFP::CreTG/^+^; TSC1^flox/flox^ (ΔTSC1) were selected for further studies. Male TSC1^flox/flox^ littermates served as control (Supplementary Fig. 3a–b). To induce Osx-Cre-directed TSC1 disruption, doxycycline was omitted from the drinking water of mice at 12 weeks of age. The ablation of TSC1 was validated by western blot analysis of tibial subchondral bone tissue (Supplementary Fig. 3c). Osterix and p-S6 double staining of tibial subchondral bone showed a dramatic increase in p-S6 level in preosteoblast cells in ΔTSC1 mice (Fig. 3a, b), indicating that mTORC1 was activated by TSC1 disruption.

Six weeks post TSC1 deletion, the subchondral bone BV/TV and BMD in ΔTSC1 mice were both remarkably increased relative to that of their control (Supplementary Fig. 3d–f). The number of Osterix^+^ cells and proliferative Osterix^+^ cells in subchondral bone were also dramatically increased, forming cell clusters on trabeculae surface (Supplementary Fig. 3g–h and Fig. 3c, d). In contrast, the number of tartrate-resistant acid phosphatase (TRAP)-positive osteoclasts remained unchanged (Supplementary Fig. 3g, i). Notably, significant aberrant bone formation and sclerosis could be observed in subchondral bone in sham ΔTSC1 mice at 6 weeks post deletion (Fig. 3e).

To further validate the role of mTORC1 activation of Osterix^+^ cells in OA development, mature male ΔTSC1 mice were subjected to ACLT and the knee joints were harvested 6 weeks post surgery. As excepted, ΔTSC1 mice developed severe sclerosis of the subchondral bone, with almost all of the marrow cavities filled with aberrant newly formed bone (Fig. 3e), and both BV/TV and BMD were increased in ACLT ΔTSC1 mice compared to that of the littermates or sham ΔTSC1 mice (Fig. 3f–h). Notably, articular cartilage degeneration was aggravated in ΔTSC1 mice following ACLT compared with that of the littermates or sham ΔTSC1 mice (Fig. 3e, i). Taken together, in the absence of surgery, ΔTSC1 mice presented subchondral bone sclerosis with little-to-no effects on articular cartilage integrity. On the other hand, ΔTSC1 mice receiving ACLT surgery exhibited more severe cartilage damage compared to that in the control and sham-operated ΔTSC1 mice. Thus, subchondral bone sclerosis driven by deletion of TSC1 in preosteoblasts is not sufficient to induce OA, but can accelerate disease progression following surgical destabilization of the joint.

### Deletion of mTORC1 in preosteoblasts prevents aberrant subchondral bone formation and OA development

**Fig. 4:**
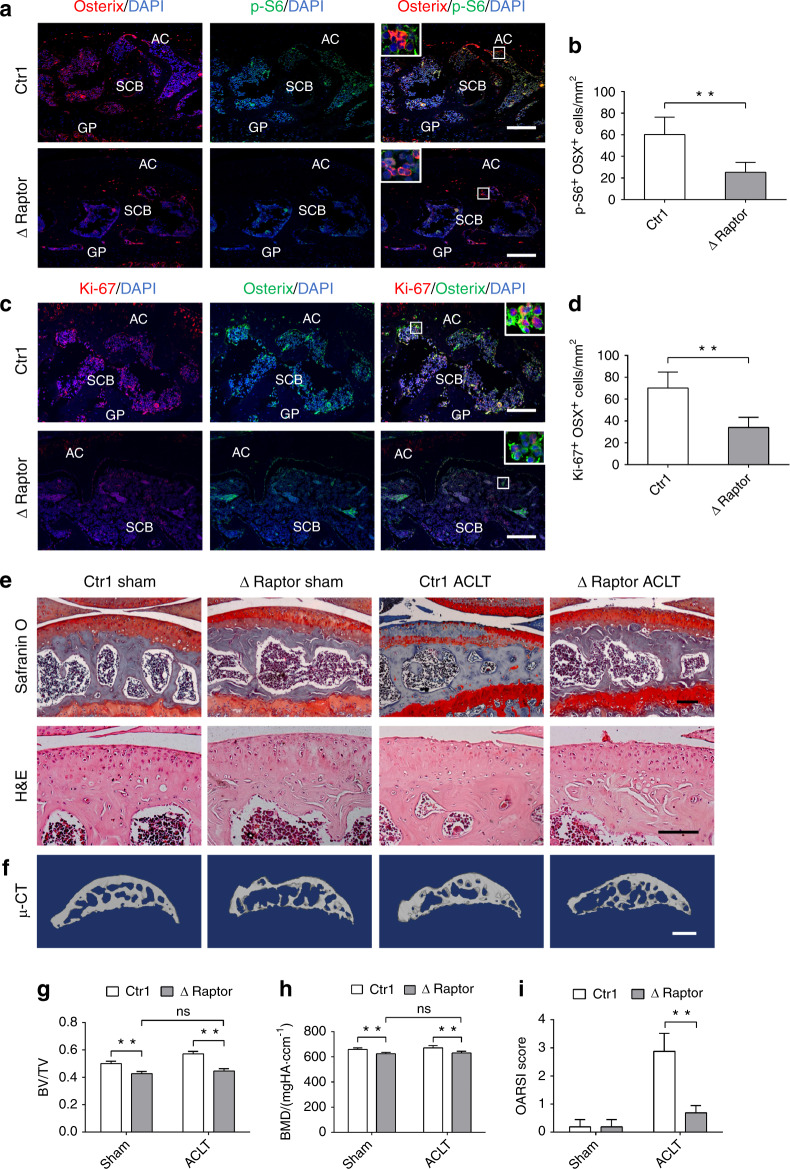
Inhibiting mTORC1 signaling in preosteoblasts prevents aberrant subchondral bone formation and OA development. **a**, **b** Representative photomicrographs and quantification of immunostaining of p-S6 in Osterix^+^ preosteoblasts in tibial subchondral bone of transgenic mice after Raptor deletion (ΔRaptor) compared to that of their littermates (Ctrl). **c**, **d** Representative photomicrographs and quantification of immunostaining of Ki-67 in Osterix^+^ preosteoblasts in tibial subchondral bone of transgenic mice after Raptor deletion (ΔRaptor) compared to that of their littermates (Ctrl). Boxed area is magnified on the top corner. Scale bars, 100 μm. AC, articular cartilage; SCB, subchondral bone; GP, growth plate. **e** Representative Safranin O-Fast green and H&E staining of sagittal sections of articular cartilage and tibia subchondral bone of ΔRaptor sham mice and ΔRaptor ACLT mice compared to that of their littermates (Ctrl). Scale bars, 100μm. **f** Representative 3D reconstructed micro-CT images of sagittal sections of tibia subchondral bone of ΔRaptor sham mice and ΔRaptor ACLT mice compared to that of their littermates (Ctrl). Scale bars, 1 mm. **g**, **h** Quantitative analysis of bone mass in subchondral bone: bone volume/total volume (BV/TV) and bone mineral density (BMD). **i** OARSI scores based on the histology analysis. Data are shown as mean±s.d. and analyzed by Student’s *t* test or two-way ANOVA . *n* ≥ 6, **P < 0.01, and ns ≥ 0.05

To further investigate the role of subchondral bone preosteoblast mTORC1 activation in OA development, we generated mice with ablation of Raptor (a key mTORC1 component) in preosteoblast cells. We crossed floxed Raptor mice with Osx1-GFP::Cre mice to generate Osx1-GFP::CreTG/^+^; Raptor^flox/flox^ (ΔRaptor) mice (Supplementary Fig. 4a–b). Doxycycline was omitted from the drinking water of mature ΔRaptor mice to delete Raptor in Osterix^+^ cells. The inactivation of mTORC1 was further confirmed by western blot and Osterix and p-S6 double staining (Supplementary Fig. 4c and Fig. 4a, b), indicating that mTORC1 was inhibited by Raptor deletion.

In contrast to mice with mTORC1 activation in preosteoblast cells, trabecular bone loss and large bone marrow cavities were observed in the subchondral bone of ΔRaptor mice with a 6-week inducible deletion (Supplementary Fig. 4d), and the BV/TV and BMD were significantly reduced (Supplementary Fig. 4e–f). The number of Osterix^+^ cells and proliferative Osterix^+^ cells in the subchondral bone marrow of ΔRaptor mice was significantly less than that of control (Supplementary Fig. 4g–h and Fig. 4c, d), with no change in the osteoclast number (Supplementary Fig. 4g and i). Notably, articular cartilage proteoglycan remained intact in the ΔRaptor mice post sham surgery (Fig. 4e).

To validate whether mice with mTORC1 inhibition in preosteoblasts could prevent subchondral aberrant bone formation and OA development, ΔRaptor mice were similarly subjected to ACLT and the knee joints were harvested at 6 weeks post surgery. Interestingly, the microarchitecture of subchondral bone was stabilized, as the BV/TV and BMD were significantly decreased in ACLT ΔRaptor mice compared with that of the littermates, but not significantly different compared with that of the sham ΔRaptor mice (Fig. 4f–h), indicating that aberrant subchondral bone formation was prevented during OA progression. Furthermore, surgically induced degeneration of articular cartilage was also attenuated relative to control mice receiving ACLT surgery (Fig. 4e, i). Taken together, these results further consolidated the theory that mTORC1 activation in Osterix^+^ cells plays a critical role in aberrant subchondral bone formation and sclerosis, and suggest that targeted inhibition of mTORC1 in subchondral Osterix^+^ cells could prevent cartilage degeneration and post-traumatic OA development.

### Activation of mTORC1 in subchondral preosteoblasts produces Cxcl12, promoting cartilage degradation

**Fig. 5:**
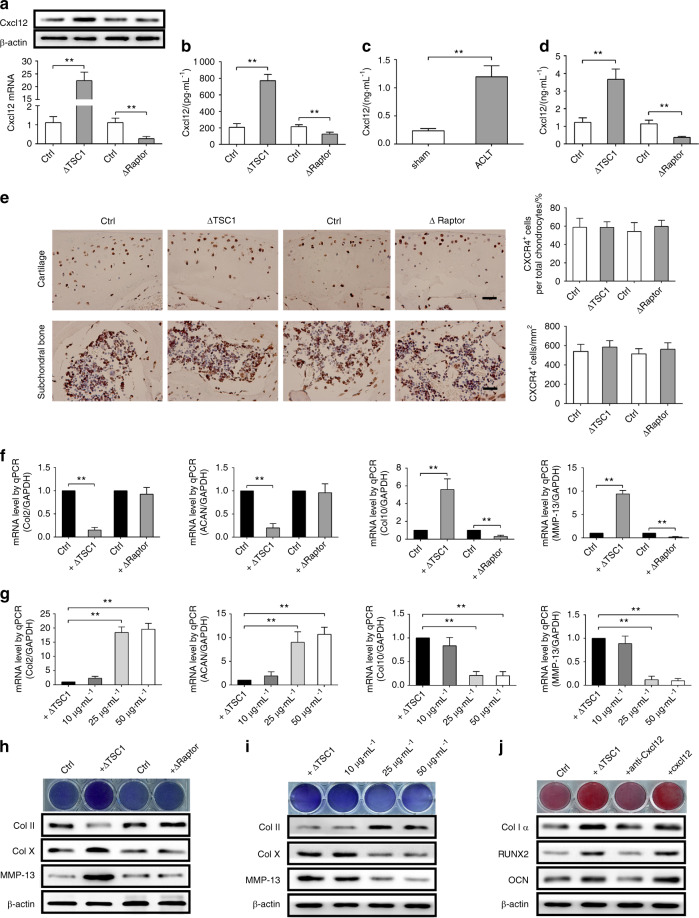
mTORC1 activation in subchondral preosteoblasts produce Cxcl12 to promote chondrocyte hypertrophic differentiation MSCs osteogenic differentiation. **a** Western blot and quantitative PCR analysis of Cxcl12 in primary preosteoblasts. **b**–**d**) Cxcl12 assessed by ELISA in the cultured medium of primary preosteoblasts or in the serum at 6 weeks post ACLT surgery. **e** Representative immunostaining and quantitative analysis of CXCR4^+^ cells in articular cartilage and subchondral bone in ΔTSC1, ΔRaptor mice, and their littermates (Ctrl). Scale bars, 100 μm. Quantitative analysis of CXCR4^+^ cells out of total cartilage chondrocytes. **f**, **g** Quantitative PCR analysis of Col2, ACAN, Col X, MMP-13 mRNA in ADTC5 cells treated with the cultured medium of primary preosteoblasts and Cxcl12-neutralizing antibody for 7 days. **h**, **i** Western blot analysis of Col2, Col X, MMP-13, and Toluidine blue staining in ADTC5 cells treated with CM of primary preosteoblasts and Cxcl12-neutralizing antibody for 7 days. **j** Western blot analysis of Col 1α, Col X, MMP-13, and Alizarin red staining in ADTC5 cells treated with CM of primary preosteoblasts, recombinant murine Cxcl12 or Cxcl12-neutralizing antibody for 14 days. Data are shown as mean ± s.d. and analyzed by Student’s *t* test and one-way ANOVA . *n* ≥ 8, **P < 0.01

**Fig. 6:**
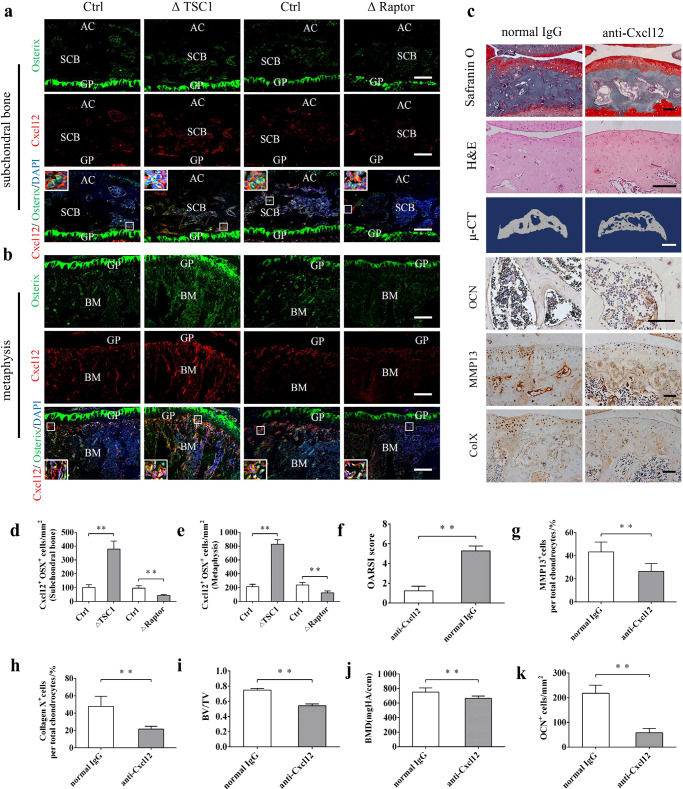
Administration of anti-Cxcl12 antibody attenuated subchondral bone remodeling and articular cartilage degeneration in ACLT mice. **a**, **b**, **d**, **e** Representative immunostaining and quantitative analysis of Cxcl12 in Osterix^+^ preosteoblasts in the subchondral bone and metaphysis in ΔTSC1, ΔRaptor mice, and their littermates (Ctrl). Boxed area is magnified on the corner. Scale bar, 100 μm. AC, articular cartilage; SCB, subchondral bone; GP, growth plate; BM, bone marrow. **c** Representative Safranin O-Fast green, H&E staining, 3D reconstructed μ-CT images and immunohistochemical staining of OCN, type X collagen, and MMP-13 in ΔTSC1 mice administered with anti-Cxcl12 antibody or normal IgG for 6 weeks. Scale bar, 100 μm (Safranin O, H&E staining, OCN, type X collagen, and MMP-13), 1 mm (μ-CT). **f** OARSI scores based on the histological analysis. **g**, **h** Quantitative analysis of type X collagen and MMP-13 in articular cartilage. **i**, **j** Quantitative analysis of bone mass in subchondral bone: bone volume/total volume (BV/TV) and bone mineral density (BMD). **k** Quantitative analysis of OCN^+^ osteoblasts in tibial subchondral bone. Data are shown as mean±s.d. and analyzed by Student’s *t* test. *n* ≥ 6, **P < 0.01

To explore the mechanisms through which subchondral mTORC1 in preosteoblasts promotes OA, we created a global messenger RNA (mRNA) expression profile of TSC1-defected or control preosteoblasts using a microarray. Among the up-regulated mRNAs, Cxcl12 has been reported to play a role in cartilage degeneration. We found that Cxcl12 was expressed constitutively in preosteoblast cells in vitro (Fig. 5a). We further confirmed the increase (>20-folds) of Cxcl12 mRNA in primary ΔTSC1 preosteoblasts, but found a decrease (>5-folds) of Cxcl12 mRNA in ΔRaptor cells versus control cells (Fig. 5a). Cxcl12 protein levels exhibited similar changes in cultured preosteoblasts. Moreover, activation of mTORC1 promoted Cxcl12 expression and inactivation of mTORC1 reduced Cxcl12 expression in Osterix^+^ preosteoblasts both in subchondral bone and primary spongiosa in metaphysis (Fig. 6a, b, d, e). Furthermore, the secretion of Cxcl12 in culture supernatant of primary ΔTSC1 preosteoblasts was up-regulated, while its secretion in ΔRaptor cells was down-regulated compared with that of control cells (Fig. 5b). Interestingly, serum Cxcl12 level was significantly increased in wild-type mice post ACLT surgery (Fig. 5c), and further increased in ACLT ΔTSC1 mice, but significantly decreased in ACLT ΔRaptor mice (Fig. 5d). Altogether, these findings demonstrate that mTORC1 positively regulates Cxcl12 expression and secretion in preosteoblasts, implying that changes in Cxcl12 level caused by mTORC1-expressing preosteoblasts may contribute to the phenotypes of articular cartilage degeneration in ΔTSC1 and ΔTSC1 ACLT mice described above.

We next sought to determine whether Cxcl12 was responsible for articular cartilage degeneration during OA. Interestingly, CXCR4, a Cxcl12 receptor, was constitutively expressed in articular cartilage chondrocytes of ΔTSC1, ΔRaptor mice, and their respective control (Fig. 5e). We further treated ACLT ΔTSC1 mice with anti-Cxcl12 antibody to neutralize endogenous Cxcl12 in vivo. Notably, articular cartilage degeneration was significantly attenuated by administration of the antibody compared to those treated with normal immunoglobulin G (IgG) (Fig. 6c, f). Moreover, the percentages of MMP-13^+^ and type X collagen^+^ chondrocytes were significantly reduced (Fig. 6c, g, h), indicating protection from cartilage degeneration. Consistently, ADTC5 cells maintained in conditioned medium (CM) from ΔTSC1 preosteoblasts exhibited hypertrophic differentiation and mineralization, compared with those cultured in CM from ΔRaptor and control preosteoblasts (Fig. 5f, h). The hypertrophic differentiation of ATDC5 cells induced by CM from TSC1-deficient preosteoblasts was significantly attenuated by Cxcl12-neutralizing antibody (Fig. 5g, i). These data suggest that the elevated Cxcl12 in preosteoblasts contributes to articular cartilage degeneration in TSC1-deficient ACLT mice.

Interestingly, CXCR4 was also detected in mesenchymal stem cells (MSCs) (Fig. 5e), and MSCs maintained in CM from ΔTSC1 preosteoblasts or supplemented with Cxcl12 exhibited osteogenic differentiation and mineralization, compared with those cultured in CM from control preosteoblasts (Fig. 5j). The effect of TSC1-deficient preosteoblasts CM on ATDC5 cells was significantly attenuated by Cxcl12-neutralizing antibody (Fig. 5j). Moreover, alleviative bone formation and bone sclerosis were observed in the subchondral bone by administration of anti-Cxcl12 antibody (Fig. 6c), as well as evidenced by decreased OCN^+^ cells, BV/TV, and BMD compared to those treated by normal mouse IgG (Fig. 6i–k). These data suggested that Cxcl12 may contribute to aberrant subchondral bone remodeling during OA development, in addition to articular cartilage degeneration.

## Discussion

Although the contribution of abnormal subchondral bone remodeling to articular cartilage degeneration and OA progression has been established,^34^ the precise mechanism by which aberrant subchondral bone formation is initiated and induces cartilage degeneration during OA is still unclear. Previously, it was shown that subchondral bone-derived tumor growth factor-β and hypoxia-inducible factor-1α could be released, causing uncoupling of subchondral bone remodeling and damage to articular cartilage in destabilized OA mouse models.^1,35,36^ These studies demonstrate that subchondral bone changes may be more sensitive to alterations in mechanical loading, and reveal the contribution of subchondral bone changes to articular cartilage degeneration in OA progression; however, the cellular and molecular mechanisms of such communication are not well defined. In the present study, we show the critical role of mTORC1 signaling by preosteoblasts in subchondral bone formation, and cross-talk to articular cartilage degeneration mediated by Cxcl12 during post-traumatic OA development. Inactivation of mTORC1 in subchondral bone preosteoblasts or treatment with Cxcl12 antibody is able to prevent subchondral bone sclerosis and attenuate articular cartilage degeneration in OA mice. Therefore, this study, the first report on mTORC1 signaling in aberrant subchondral bone formation and the interplay between subchondral bone and articular cartilage during OA progression, shows that targeted inhibition of preosteoblast mTORC1 provides a novel prospective therapy for OA.

Both clinical and animal studies have reported that progression of OA is accompanied by turnover in the subchondral bone microarchitecture.^10,37^ Aberrant subchondral bone formation and sclerosis have been reported in human OA joints^38^ and in several experimental OA models.^39^ Similarly, we found that the subchondral bone underwent dramatic remodeling after surgery in ACLT mice and OA patients. The identified risk factors for OA, including ligament injury, excessive body weight, and muscle weakening during aging, increase the mechanical loading on weight-bearing joints.^40–42^ The architecture of subchondral bone and plate adapt via modeling and remodeling in response to these mechanical stresses. Because the subchondral bone acts as a structural girder and shock absorber for the adjacent articular cartilage, aberrant bone formation and sclerosis enhance the subchondral bone holding strength and stiffness, but reduce elastic deformation and shock absorption capacity, causing an imbalance in mechanical stress distribution and/or excessive loading in articular cartilage, which in turn causes articular cartilage wear as a result of joint motion.^35,43^ Preventing aberrant bone formation and sclerosis in subchondral bone is a promising therapeutic strategy to curb the progression of OA.

mTORC1 is known for its anabolic effects on articular cartilage homeostasis by stimulating the production of extracellular matrix proteins and preventing terminal differentiation of chondrocytes,^23^ but the role of mTORC1 in subchondral bone changes during OA is unclear. In this study, we observed that changes in mechanical loading increased mTORC1 activity in subchondral bone preosteoblasts in mice and in vitro. In addition, mTORC1 is also activated in subchondral bone preosteoblasts from OA patients. As we and other groups have shown that mTORC1 activation in Osterix^+^ cells strongly promotes preosteoblast proliferation and expansion and produces high bone mass in mice,^25,44^ mTORC1 activation may stimulate subchondral preosteoblast proliferation and sustained subchondral bone formation in ACLT and ΔTSC1 mice. Notably, during the normal remodeling process, osteoblasts and their progenitors are located at resorption sites on the bone surface. However, abnormal mechanical loading-induced mTORC1 activation of subchondral preosteoblasts promotes their proliferation and “in situ” formation of preosteoblast clusters in the bone marrow cavities. We suggest that these clustered preosteoblasts, spatiotemporally uncoupled from osteoclastic bone resorption, produce large amount of less mineralized bone in the bone marrow, leading to subchondral bone sclerosis. These data reveal that mTORC1 plays an essential role in aberrant subchondral bone formation and sclerosis during OA development.

Previous studies have shown that Cxcl12 is a catabolic factor that can infiltrate cartilage, reduce proteoglycan content, and increase MMP-13 activity.^45^ Inhibiting the Cxcl12/CXCR4 signaling pathway can attenuate the pathogenesis of OA.^46^ Moreover, it also induces the migration of bone marrow MSCs to articular lesion sites. For the first time, we established the existence of cross-talk between bone-forming preosteoblasts and articular chondrocytes mediated by Cxcl12. Cxcl12 was expressed constitutively in preosteoblasts and regulated by mTORC1. Furthermore, serum Cxcl12 level was drastically up-regulated by mTORC1 activation of preosteoblasts during OA, which was detected in ACLT mice. Its receptor CXCR4 was expressed in articular cartilage chondrocytes. We speculated that a direct effect of Cxcl12 on these cells might contribute to articular cartilage degeneration observed in our mouse models. Indeed, chondrocytes and preosteoblasts co-cultured in vitro generated similar effects to those observed in articular cartilage when exposed directly to Cxcl12 in vivo. Importantly, Cxcl12-neutralizing antibody administration reduced cartilage degeneration and alleviated OA development in OA and mutant mice. These results support our conclusion that Cxcl12 mediates articular cartilage degeneration stimulated by mTORC1 activation in subchondral preosteoblasts during OA.

In order to investigate the role of mTORC1 activation in subchondral bone remodeling during post-traumatic OA development, the ACLT OA mouse model was employed in this study according to previous studies.^1,47^ However, ACLT model might be too severe to represent human OA. The DMM model is more reliable, reproducible, and structural similar to human degenerative OA.^48^ The mTORC1 activation in the DMM model is of interest for further investigation.

**Fig. 7:**
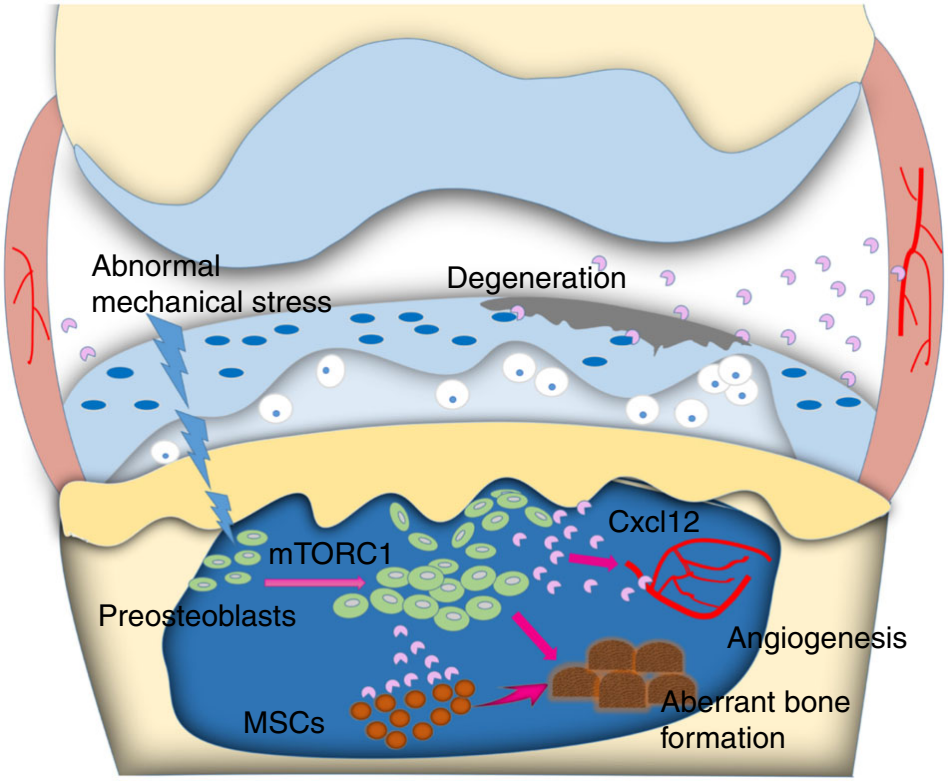
Model of elevated active mTORC1 in the subchondral bone at the onset of OA. mTORC1 is activated in the subchondral bone in response to abnormal mechanical loading, which induces preosteoblast abnormal proliferation and Cxcl12 secretion to promote subchondral bone formation and articular cartilage degeneration for OA progression

Taken together, the results of this study suggest a novel pathological mechanism for OA development on subchondral bone–articular cartilage interplay. Abnormal mechanical loading activates mTORC1 in subchondral bone preosteoblasts in OA, and mTORC1 activation promotes preosteoblast proliferation and Cxcl12 secretion, resulting in aberrant subchondral bone formation and bone sclerosis, and articular cartilage degeneration (Fig. 7). Consequently, pharmaceutical inhibition of the pathways might be beneficial for OA patients.

## Materials and methods

### Human samples

After the study was approved by the Ethics Committee of the Third Affiliated Hospital of Southern Medical University, 38 tibial plateaus (including articular cartilage) were obtained from OA patients who were undergoing knee replacement surgery. Eight normal samples from victims of road traffic accidents with no history of arthritic diseases served as control. The specimens were processed for micro-CT analysis, histological examination, and calculated per patient in three sequential sections with OARSI scoring system as previously described.^49^ The number of positively stained cells in the tibia subchondral bone area was counted in three sequential sections per patient in each group.

### Mice

We purchased the Tsc1^flox/flox^, Raptor^flox/flox^, R26-mT/mG, and Osx1-GFP::Cre mouse strains from the Jackson Laboratory (Bar Harbor, ME, USA). The background of Tsc1^flox/flox^ mice is 129S4/SvJae, and these mice were backcrossed to mice with a C57BL/6 background for eight generations before use. We performed genotyping using genomic DNA isolated from tail biopsies, using the following primers: loxP Tsc1 allele forward, 5ʹ-GTCACGACCGTAGGAGAAGC-3ʹ and reverse, 5ʹ-GAATCAACCCCACAGAGCAT-3ʹ; loxP Raptor allele forward, 5ʹ -CTCAGTAGTGGTATGTGCTCAG-3ʹ and reverse, 5ʹ-GGGTACAGTATGTCAGCACAG-3ʹ; Osx1-GFP::Cre forward, 5ʹ-CTCTTCATGAGGAGGACCCT-3ʹ and reverse, 5ʹ-GCCAGGCAGGTGCCTGGACAT-3ʹ.

We purchased C57BL/6J (wild-type) mice from the Experimental Animal Centre of Southern Medical University (Guangzhou, China). Importation, transportation, housing, and breeding of the mice were all conducted according to the recommendations of “The use of non-human primates in research.” The mice were euthanatized by cervical dislocation to prevent suffering. The Southern Medical University Animal Care and Use Committee approved all procedures involving the mice.

### Doxycycline treatment

Osx-Cre (Osx1-GFP::Cre), a BAC transgenic mouse line expressing a GFP::Cre fusion protein from the regulatory sequence of Osx, was generated to direct gene deletion in the osteoblast lineage.^50^ The characterization of this mouse line revealed that Cre activity is largely restricted to the osteogenic perichondrium, periosteum, and osteoblast-lineage cells within the marrow cavity.^51^ Cre-mediated recombination in these mice is under the control of doxycycline. To prevent the Osx promoter from driving Cre expression, mated mice were fed with doxycycline (Sigma-Aldrich, St. Louis, MO, USA; 9891) in drinking water to deliver a daily dose of 2–3 mg/mouse of doxycycline according to the manufacturer’s instruction. Cre activity was indeed suppressed in Osx-Cre mice by doxycycline treatment in Osx-Cre; R26-mT/mG mice (Supplementary Fig. 2d).

### Experimental OA model, anti-Cxcl12 antibody treatment, and histomorphometry

Three-month-old male C57BL/6J, ΔTSC1, ΔRaptor mice and control mice were subjected to ACL transection (ACLT) surgical procedure to induce mechanical instability-associated OA, as previously described,^52^ and the procedure to establish sham operation group was similar to operation group but without transecting the ACL. Knee joints freshly dissected from mice were fixed for 48 h in 4% paraformaldehyde at 4 °C, and then decalcified for 21 days in 10% EDTA (pH 7.4). The tissues were embedded in paraffin and sectioned continuously (4 μm thick) and serial sections were obtained from the medial and lateral compartments at 50-μm intervals. Eight representative mid-sagittal sections were deparaffinized in xylene, hydrated with graded ethanol, and stained with H&E or Safranin O/Fast Green. We quantified the degeneration of the articular cartilage of the medial and lateral tibial plateau joint with the OARSI scoring system as previously described.^53^ The final score was generated by averaging individual scores.

### Micro-CT analysis

The knee joint specimens were analyzed by high resolution μ-CT Scanner (Viva CT80; Scanco Medical AG, Bassersdorf, Switzerland). The scanner was set at a voltage of 55 kV, a current of 145 mA and a resolution of 10 μm per pixel. Cross-sectional images of the tibial subchondral bone were used to perform three-dimensional histomorphometric analysis. We defined the region of interest to cover the whole subchondral bone medial compartment, and we used a total of ten consecutive images from medial tibial plateau for 3D reconstruction and analysis. Three-dimensional structural parameters analyzed including: bone mineral density (BMD), BV/TV, trabecular number and trabecular thickness.

### Immunohistochemical and immunofluorescent analyses

TRAP staining (Sigma-Aldrich, Missouri, USA) and immunostaining were performed according to the manufacturer’s instructions. Serial sections were incubated with primary antibodies to anti-Osterix (Abcam, ab22552; Santa Cruz, sc-393060), anti-Ki-67 (Immunoway, YM-3064), anti-p-S6 ribosomal protein (Ser235/236) (Cell Signaling Techonlogy, #2211), anti-Cxcl12 (Abcam, ab9797), anti-CXCR4 (Abcam, ab124824), anti-collagen X (Abcam, ab58632), and anti-MMP-13 (Abcam, ab39012) overnight at 4 °C. For immunofluorescent staining, secondary antibodies conjugated with fluorescent tags were added and slides were incubated at room temperature for 1h in dark. Photomicrographs of sections were captured to perform histomorphometric measurements on the entire area of the tibial subchondral bone with ZEISS Scope A1 (Zeiss, Heidelberg, Germany), Quantitative histomorphometric analysis was conducted in a blinded fashion using the OsteoMeasure XP Software (OsteoMetrics, Inc., Atlanta, USA). The number of positively stained cells in the entire tibia subchondral bone area was counted in three sequential sections per mouse in each group.

### Cell culture

The chondrogenic cell line ATDC5 (Riken BioResource Center, Tsukuba, Japan) was cultured in maintenance medium consisting of Dulbecco’s modified Eagle’s medium (DMEM) nutrient mixture F12 (DMEM:F12) (Gibco) supplemented with 5% fetal bovine serum (FBS) (Gibco). Chondrogenesis of ATDC5 cells was induced by treatment with insulin, transferrin, and selenous acid (Sigma-Aldrich) according to a previously reported method.^54^ Bone marrow-mesenchymal stromal cells were isolated from bone marrow of C57BL/6 mice and previously characterized by phenotyping and trilineage differentiation potential as described previously.^55^ They were expanded in proliferative medium consisting in DMEM, 100 μg·mL^−1^ penicillin/streptomycin, 2 mmol·L^−1^ glutamine, and supplemented with 10% FBS. For osteogenic induction, 50 μg·mL^−1^ l-ascorbic acid (Sigma-Aldrich) and 10 mmol·L^−1^ β-glycerol phosphate (Sigma-Aldrich) were added.

Primary preosteoblast cells were isolated from the calvaria of newborn mice. In brief, calvariae were dissected from the mice (24h after birth), rinsed with phosphate-buffered saline (PBS) and digested in freshly prepared 0.1 mg·mL^−1^ collagenase type-II (Thermo Fisher Scientific, Waltham, MA, USA) in α-minimal essential Eagle’s medium (α-MEM) at 37 °C for 20 min; the digestion was repeated five times. After digestion, supernatants were combined and centrifuged to pellet the cells.^56^ Cells were then maintained in α-MEM (Gibco, Thermo Fisher Scientific) supplemented with 10% FBS (Gibco), 100 U·mL^−1^ penicillin and 100 mg·mL^−1^ streptomycin sulfate, at 37 °C with 5% CO_2_. After reaching confluence in 60-mm culture dishes, the medium was replaced with α-MEM (Gibco) supplemented with 1% bovine serum albumin, and the cells were cultured for 16 h before collecting the CM. The CM was then centrifuged for 15 min at 1 500 × *g* at 4 °C, and then stored at −80 °C until use. Mouse Cxcl12 antibody (R&D Systems, Minneapolis, MN, USA, #MAB310, 10–50 μg·mL^−1^) and recombinant murine Cxcl12 (PrimeGene Bio-Tech, #20315, 100 ng·mL^−1^) was added to the CM as indicated.

### Toluidine blue staining and Alizarin red staining

Cultured cells were fixed with 4% paraformaldehyde for 30 min at room temperature, then stained using a Toluidine Blue Staining Kit (Leagene, Beijing, China) for 60 min at room temperature, and finally washed with PBS to remove excess dye.

For the detection of osteogenic differentiation, the Alizarin red assay (Sigma-Aldrich) was performed to determine mineralization. Briefly, cells were washed with PBS, fixed with paraformaldehyde for 30 min, incubated with 1% Alizarin red for 30 min at room temperature, and washed with PBS to remove the excess of staining. Osteogenic nodules were stained in orange-red due to calcium deposition.

### ELISA analysis

We used the mouse (SDF1) ELISA (enzyme-linked immunosorbent assay) Kit (Elabscience Biotechnology Co. Ltd, Wuhan, China; #E-EL-M1094c) to analyze Cxcl12 in serum and CM. ELISA analysis was performed according to the manufacturer’s instructions.

### Real-time quantitative PCR and microarray analysis

Total RNA was isolated from cell pellets using TRIzol reagent (Life Technologies, Grand Island, NY, USA). Complementary DNA was reversely transcribed from RNA samples using reverse transcription reagents (Vazyme Biotech Co. Ltd, Nanjing, China) and quantitative PCR assays were carried out to quantify levels of mRNA expression of Cxcl12, type-II collagen (Col2), aggrecan (ACAN), type X collagen (Col10), and collagenolytic MMP (MMP-13) with glyceraldehyde 3-phosphate dehydrogenase (GAPDH) as the internal loading control using Real-Time PCR Mix (Vazyme Biotech Co. Ltd) in a Light Cycler (Roche Molecular Biochemicals, Indianapolis, IN, USA). The following primer sequences were used: Cxcl12 (forward primer: 5′-TCCCCTTGTGTTTTGGCAGT-3′; reverse primer: 5′-TTGCATCTCCCACGGATGTC-3′); Col2 (forward primer: 5′-CACACTGGTAAGTGGGGCAAGACCG-3′; reverse primer: 5′-GGATTGTGTTGTTTCAGGGTTCGGG-3′); ACAN (forward primer: 5′-GAAGGTGAAGGTCGGAGTC-3′; reverse primer: 5′-GAAGATGGTGATGGGATTTC-3′); Col10 (forward primer: 5′-AAGTGGACCGAAAGGAGACA-3′; reverse primer: 5′-TGGAAACCCATTCTCACCTC-3′); MMP-13 (forward primer: 5′-GCTGCGGTTCACTTTGAGAA-3′; reverse primer, 5′-GGCGGGGATAATCTTTGTCCA-3′); and GAPDH (forward primer: 5′-AAATGGTGAAGGTCGGTGTGAAC-3′; reverse primer, 5′- CAACAATCTCCACTTTGCCACTG-3′).

For mRNA array analysis, samples were submitted to Shanghai Biotechnology Corporation for hybridization on an Agilent-014868 Whole Mouse Genome Microarray 4×44K G4122F (Probe Name version). Each microarray chip was hybridized to a single sample labeled with Cy3. Background subtraction and normalization were performed. Finally, mRNAs with expression levels differing by at least 3-folds between control and TSC1-defected preosteoblasts were selected (*P*<0.05). Microarray data have been deposited in the GEO database under accession code GSE74781.

### Western blot analysis

Cells were lysed with 2% sodium dodecyl sulfate (SDS), 2 mol·L^−1^ urea, 10% glycerol, 10 mmol·L^−1^ Tris-HCl (pH 6.8), 10 mmol·L^−1^ dithiothreitol, and 1 mM phenylmethylsulfonyl fluoride. The lysates were centrifuged and the supernatants were separated by SDS-polyacrylamide gel electrophoresis and blotted onto a nitrocellulose membrane (Bio-Rad Laboratories, Hercules, CA, USA). The membrane was then incubated with specific antibodies to phospho-S6 (S235/236) (Cell Signaling Techonlogy, #2211), S6 (Santa Cruz Biotechnology Inc., sc-74459), phospho-AMPK (Thr172) (Cell Signaling Technology, #2535), AMPK (Cell Signaling Technology, #2532), Cxcl12 (Abcam, ab9797), collagen X (Abcam, ab58632), MMP-13 (Abcam, ab39012), collagen II (Abcam, ab185430), collagen I (Abcam, ab6308), osteocalcin (Santa Cruz Biotechnology Inc., sc-23790), RUNX2 (Abclonal, A2851), and β-actin (Cell Signaling Technology, #3700). The membrane was then visualized using an Enhanced Chemiluminescence Kit (Amersham Biosciences, Chalfont St. Giles, UK).

### Statistical analysis

All results were presented as the mean±s.d. using GraphPad Prism 6.00 (GraphPad Software, San Diego, CA, USA) and analyzed using Student’s *t* test or ANOVA (analysis of variance). Pearson’s linear correlation coefficients were used to measure the dependency of two variables. The level of significance was set at *P* < 0.05.

## Electronic supplementary material

Supplemental Figure legends and Figure
