# Correction to: Activation of mTORC1 in subchondral bone preosteoblasts promotes osteoarthritis by stimulating bone sclerosis and secretion of CXCL12

**DOI:** 10.1038/s41413-019-0065-8

**Published:** 2019-08-28

**Authors:** Chuangxin Lin, Liangliang Liu, Chun Zeng, Zhong-Kai Cui, Yuhui Chen, Pinling Lai, Hong Wang, Yan Shao, Haiyan Zhang, Rongkai Zhang, Chang Zhao, Hang Fang, Daozhang Cai, Xiaochun Bai

**Affiliations:** 1 Department of Orthopedics, Academy of Orthopedics-Guangdong Province, The Third Affiliated Hospital of Southern Medical University, 510630 Guangzhou, China; 2 Key Laboratory of Mental Health of the Ministry of Education, Department of Cell Biology, School of Basic Medical Sciences, Southern Medical University, 510515 Guangzhou, China; 3 Department of Orthopedic Surgery, Shantou Central Hospital, Affiliated Shantou Hospital of Sun Yat-Sen University, 515041 Shantou, China

**Correction to:** *Bone Research* 10.1038/s41413-018-0041-8, published online 20 February 2019

During re-read of our previously article^1^ published in *Bone Research*, we regretted to find a mistake in Fig. 5j figure legends and Fig. 6f, j, respectively due to the clerical errors or mislabeling in paper preparation. Although this correction does not affect the results or conclusion of the above paper, all the authors agree to correct this negligence as providing the right Fig. 5j figure legends and Fig. 6f, j presented below. We feel sorry and apologize for all the inconvenience caused.

**Figure:**
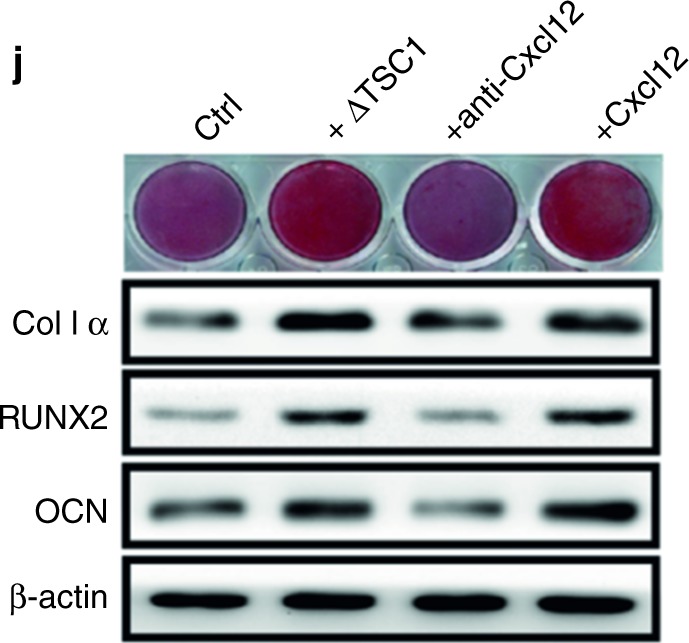
Fig. 5

**Figure:**
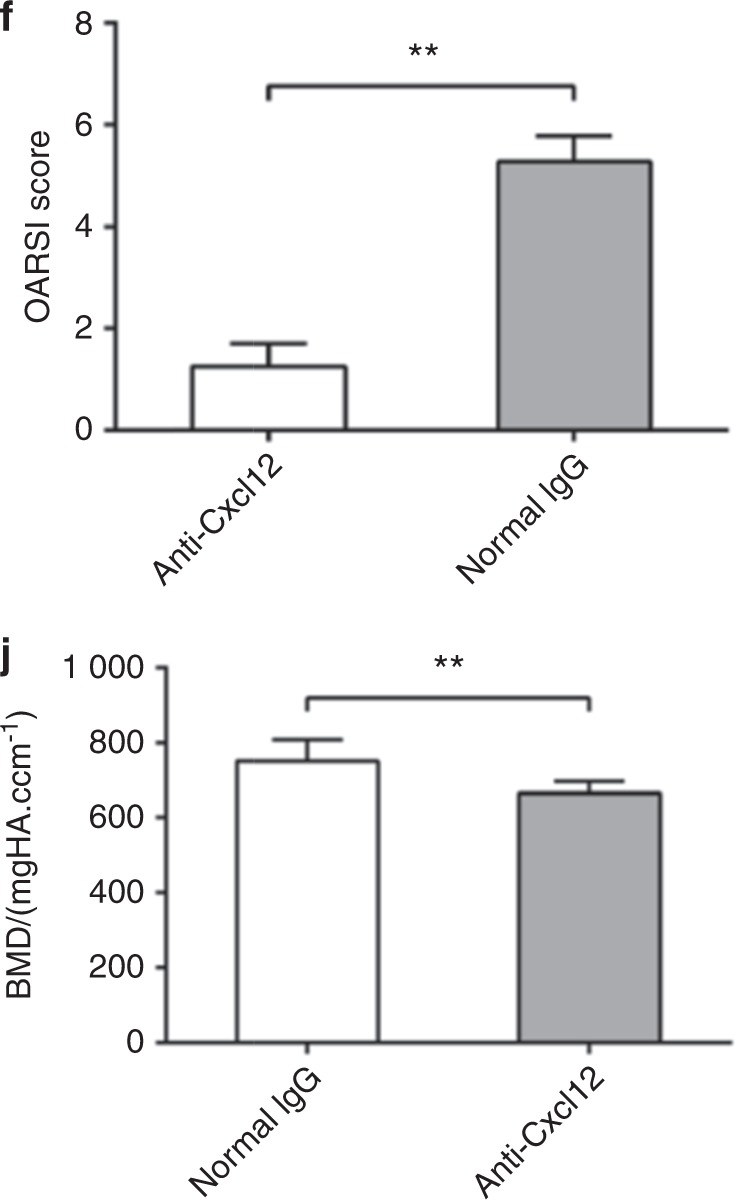
Fig. 6

These corrections are in Fig. 5j and described in the “Results” subsection “Activation of mTORC1 in preosteoblasts produces Cxc12, promoting cartilage degradation” and it reads, “...The effect of TSC1-deficient preosteoblasts CM on *MSCs* was significantly attenuated by Cxcl12-neutralizing antibody (Fig. 5j).”

Accordingly in Fig. 5j figure legends, the correction reads “...j Western blot analysis of Col 1α, RUNX2, OCN, and Alizarin red staining in *MSCs* treated with CM of primary preosteoblasts, recombinant murine Cxcl12 or Cxcl12-neutralizing antibody for 14 days.”

The correction to Fig. 6f, j.

The Supplementary Information for the “Materials and methods” subsection “Experimental OA model, anti-Cxcl12 antibody treatment, and histomorphometry ” is presented below:

“Three-month-old male C57BL/6J, ΔTSC1, ΔRaptor mice and control mice were subjected to ACL transection (ACLT) surgical procedure to induce mechanical instability-associated OA, as previously described, and the procedure to establish sham operation group was similar to operation group but without transecting the ACL. Some ΔTSC1 ACLT mice were treated with a neutralizing Cxcl12 antibody (R&D Systems, Minneapolis, MN) 50 μg/week or the same dose of normal immunoglobulin G (R&D Systems) by intraperitoneal injection for 6 weeks.”
