# Author Correction: Activation of mTORC1 in subchondral bone preosteoblasts promotes osteoarthritis by stimulating bone sclerosis and secretion of CXCL12

**DOI:** 10.1038/s41413-025-00462-9

**Published:** 2025-10-17

**Authors:** Chuangxin Lin, Liangliang Liu, Chun Zeng, Zhong-Kai Cui, Yuhui Chen, Pinling Lai, Hong Wang, Yan Shao, Haiyan Zhang, Rongkai Zhang, Chang Zhao, Hang Fang, Daozhang Cai, Xiaochun Bai

**Affiliations:** 1 Department of Orthopedics, Academy of Orthopedics- Guangdong Province, The Third Affiliated Hospital of Southern Medical University, Guangzhou, 510630 China; 2 Department of Orthopedic Surgery, Shantou Central Hospital, Affiliated Shantou Hospital of Sun Yat-Sen University, Shantou, 515041 China; 3 Key Laboratory of Mental Health of the Ministry of Education, Department of Cell Biology, School of Basic Medical Sciences, Southern Medical University, Guangzhou, 510515 China

Correction to: *Bone Research* 10.1038/s41413-018-0041-8, published online 20 February 2019

Following publication of the article,^1^ the authors detected unintentional image layout errors in the mice-12 weeks group p-S6 of Fig. 2b, the ΔTSC1group metaphysis of Fig. 6b, the normal lgG group OCN of Fig. 6c, and the ΔTSC1group osterix of supplementary Fig. 3g, which necessitate correction due to the improper use of images. These amendments are required; however, they do not alter the study's conclusions or the overall interpretation of the article.

Incorrect Fig. 2:
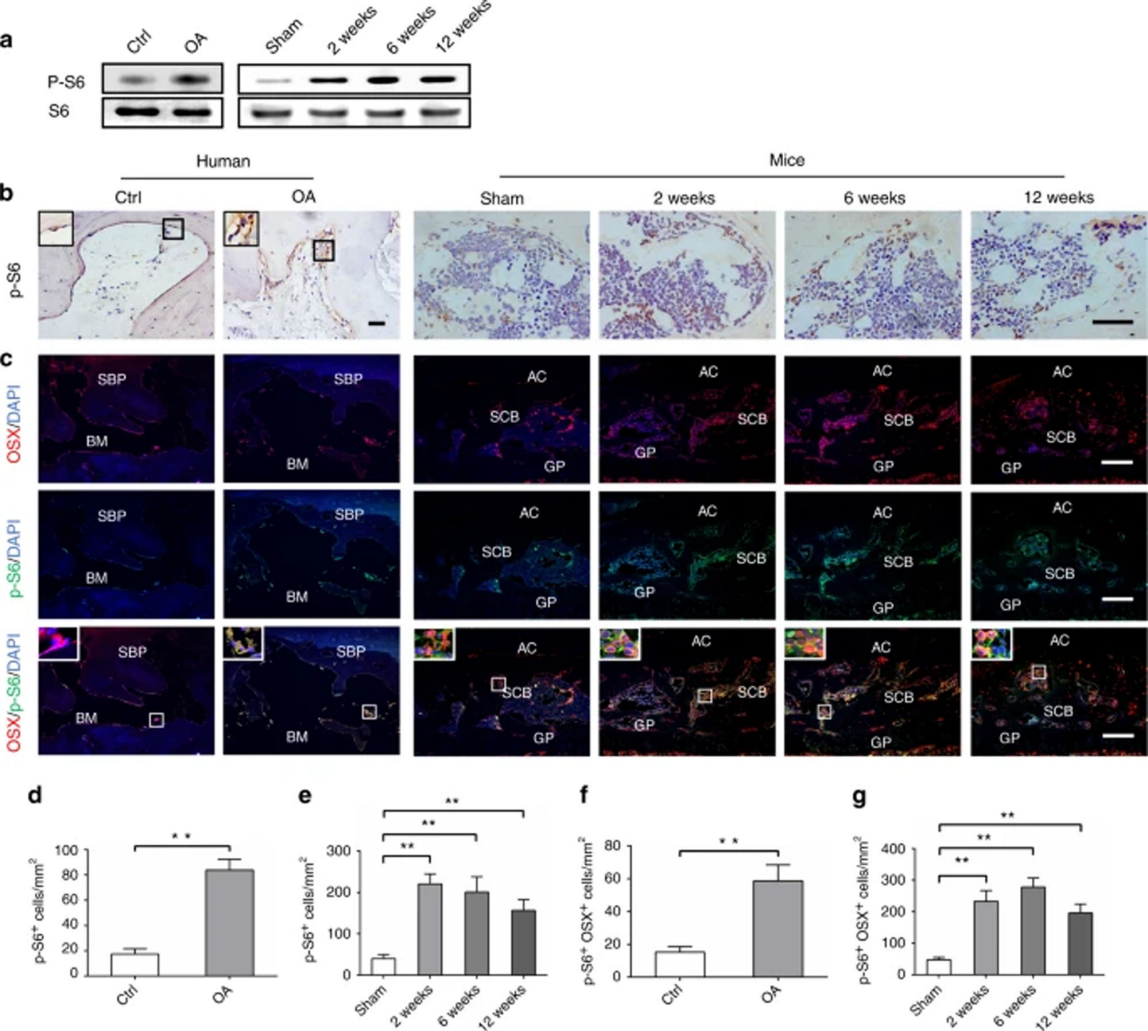


**Fig. 2** mTORC1 is activated in subchondral bone preosteoblasts in OA patients and destabilized OA mice. **a** Western blot analysis of p-S6 expression in tibia subchondral bone tissues from patients and OA mice. **b**, **d**, **e** Representative immunostaining and quantitative analysis of p-S6^+^ cells in tibial subchondral bone of OA patients and OA mice. Scale bars, 200 μm. **c**, **f**, **g** Representative immunostaining and quantitative analysis of p-S6 in Osterix^+^ preosteoblasts in tibial subchondral bone of OA patients and OA mice. Boxed area is magnified on the top left corner. Scale bars, 100 μm. SBP subchondral bone plate, BM bone marrow, AC articular cartilage, SCB subchondral bone, GP growth plate. Data are shown as mean ± s.d. and analyze by Student’s *t* test or one-way ANOVA. *n* ≥ 8, **P < 0.01

Correct Fig. 2:
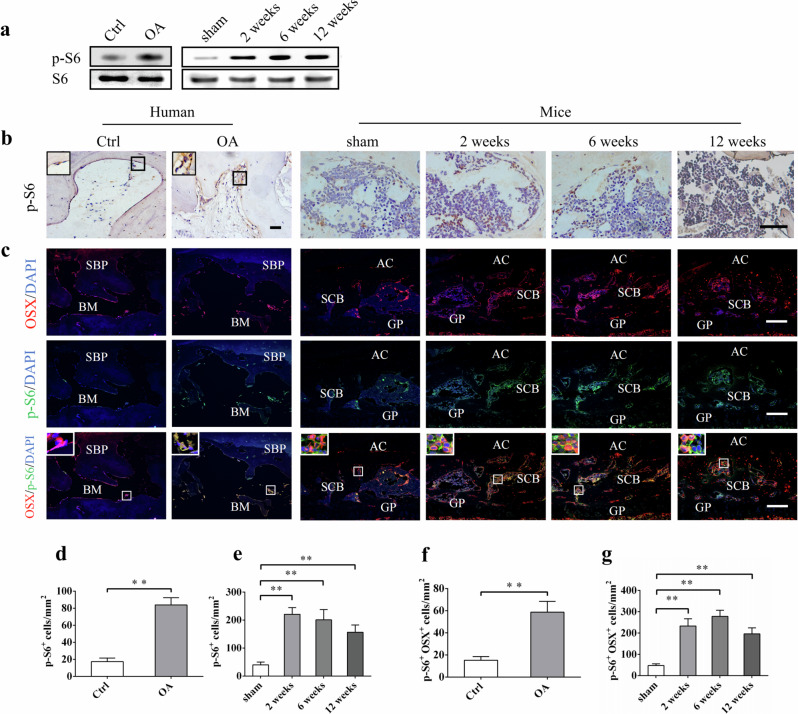


**Fig. 2** mTORC1 is activated in subchondral bone preosteoblasts in OA patients and destabilized OA mice. **a** Western blot analysis of p-S6 expression in tibia subchondral bone tissues from patients and OA mice. **b**, **d**, **e** Representative immunostaining and quantitative analysis of p-S6^+^ cells in tibial subchondral bone of OA patients and OA mice. Scale bars, 200 μm. **c**, **f**, **g** Representative immunostaining and quantitative analysis of p-S6 in Osterix^+^ preosteoblasts in tibial subchondral bone of OA patients and OA mice. Boxed area is magnified on the top left corner. Scale bars, 100 μm. SBP subchondral bone plate, BM bone marrow, AC articular cartilage, SCB subchondral bone, GP growth plate. Data are shown as mean ± s.d. and analyze by Student’s *t* test or one-way ANOVA. *n* ≥ 8, **P < 0.01

Incorrect Fig. 6:
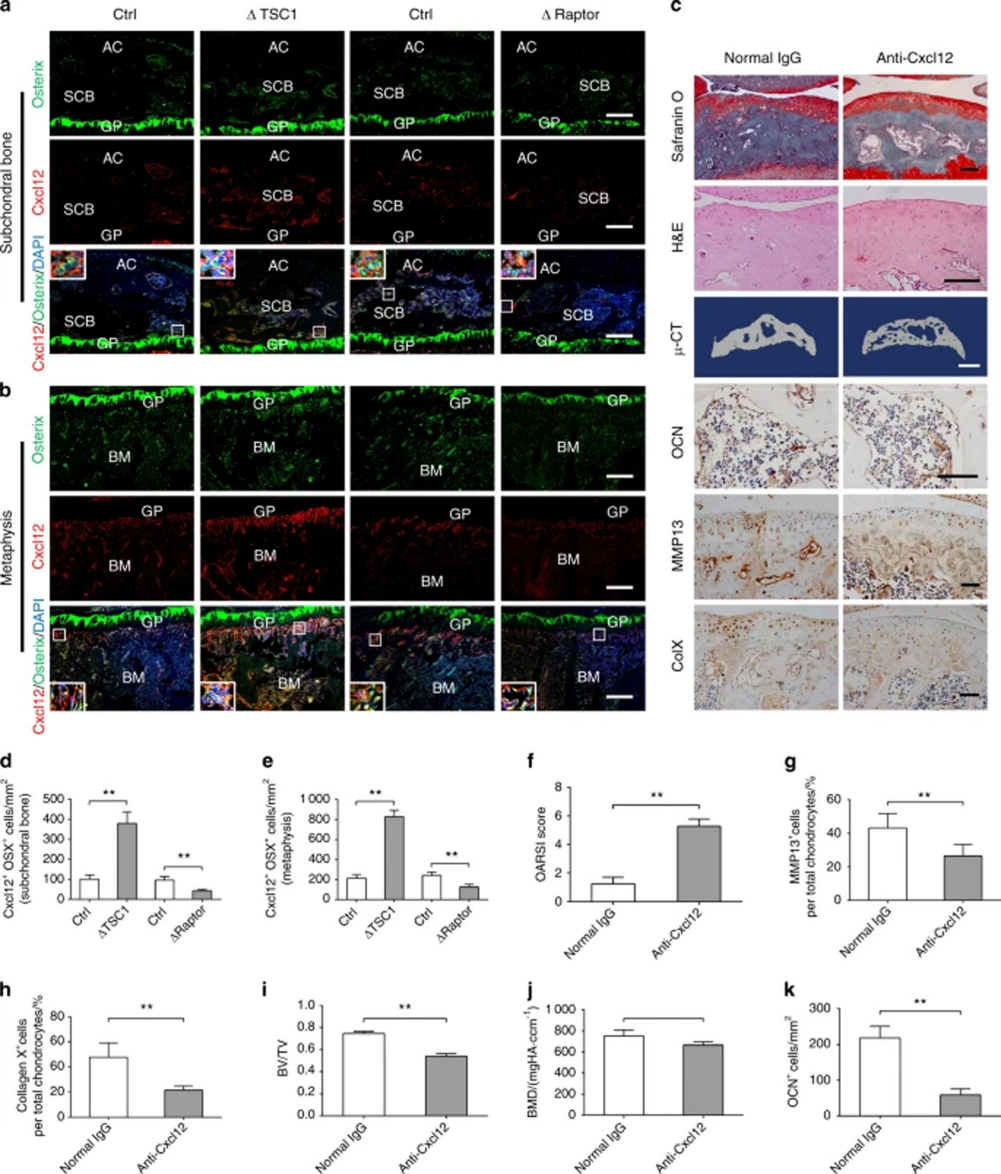


**Fig. 6** Administration of anti-Cxcl12 antibody attenuated subchondral bone remodeling and articular cartilage degeneration in ACLT mice. **a**, **b**, **d**, **e** Representative immunostaining and quantitative analysis of Cxcl12 in Osterix^+^ preosteoblasts in the subchondral bone and metaphysis in ΔTSC1, ΔRaptor mice, and their littermates (Ctrl). Boxed area is magnified on the corner. Scale bar, 100 μm. AC articular cartilage, SCB subchondral bone, GP growth plate, BM bone marrow. **c** Representative Safranin O-Fast green, H&E staining, 3D reconstructed μ-CT images and immunohistochemical staining of OCN, type X collagen, and MMP-13 in ΔTSC1 mice administered with anti-Cxcl12 antibody or normal IgG for 6 weeks. Scale bar, 100 μm (Safranin O, H&E staining, OCN, type X collagen, and MMP-13), 1 mm (μ-CT). **f** OARSI scores based on the histological analysis. **g**, **h** Quantitative analysis of type X collagen and MMP-13 in articular cartilage. **i**, **j** Quantitative analysis of bone mass in subchondral bone: bone volume/total volume (BV/TV) and bone mineral density (BMD). **k** Quantitative analysis of OCN^+^ osteoblasts in tibial subchondral bone. Data are shown as mean ± s.d. and analyzed by Student’s *t* test. *n* ≥ 6, **P < 0.01

Correct Fig. 6:
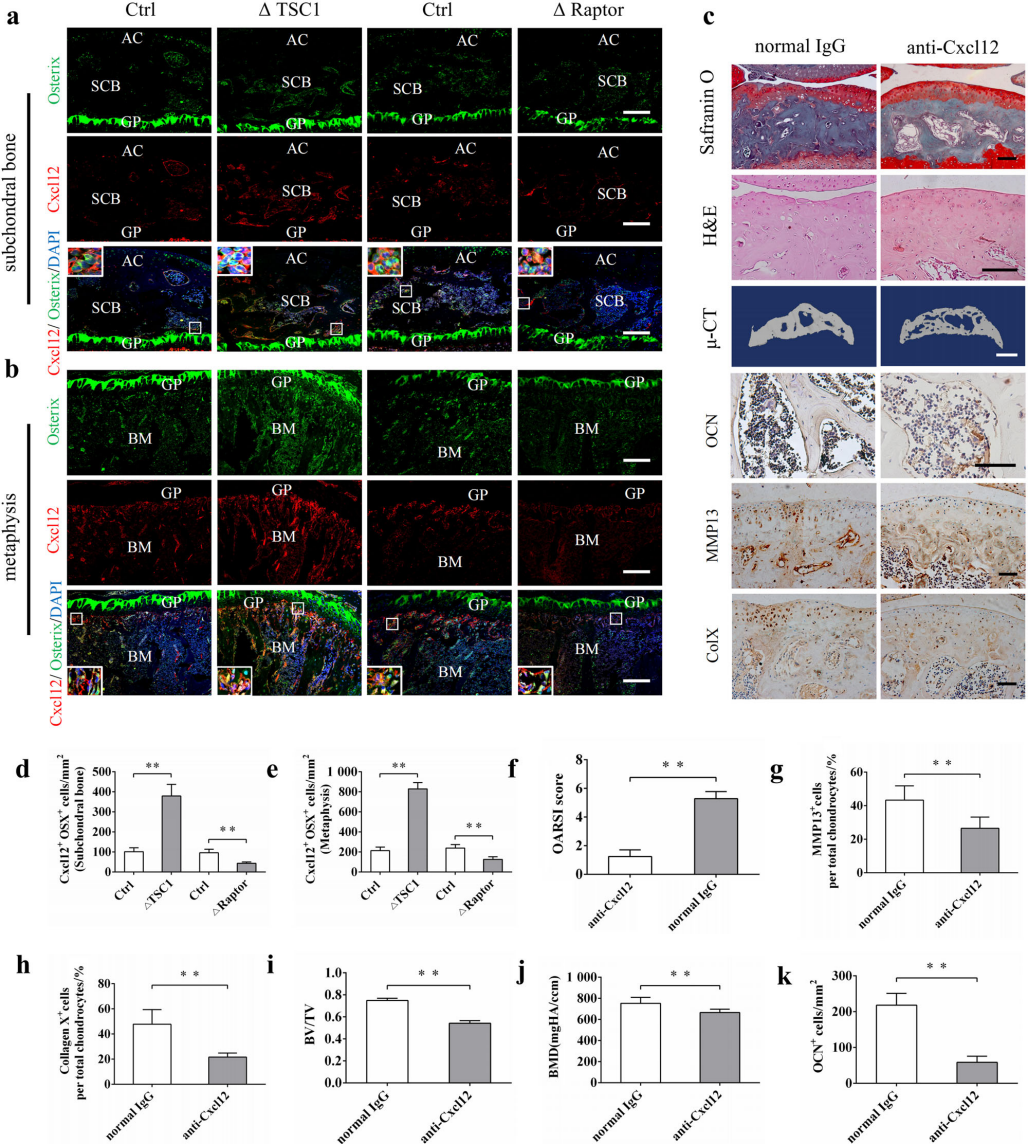


**Fig. 6** Administration of anti-Cxcl12 antibody attenuated subchondral bone remodeling and articular cartilage degeneration in ACLT mice. **a**, **b**, **d**, **e** Representative immunostaining and quantitative analysis of Cxcl12 in Osterix^+^ preosteoblasts in the subchondral bone and metaphysis in ΔTSC1, ΔRaptor mice, and their littermates (Ctrl). Boxed area is magnified on the corner. Scale bar, 100 μm. AC articular cartilage, SCB subchondral bone, GP growth plate, BM bone marrow. **c** Representative Safranin O-Fast green, H&E staining, 3D reconstructed μ-CT images and immunohistochemical staining of OCN, type X collagen, and MMP-13 in ΔTSC1 mice administered with anti-Cxcl12 antibody or normal IgG for 6 weeks. Scale bar, 100 μm (Safranin O, H&E staining, OCN, type X collagen, and MMP-13), 1 mm (μ-CT). **f** OARSI scores based on the histological analysis. **g**, **h** Quantitative analysis of type X collagen and MMP-13 in articular cartilage. **i**, **j** Quantitative analysis of bone mass in subchondral bone: bone volume/total volume (BV/TV) and bone mineral density (BMD). **k** Quantitative analysis of OCN^+^ osteoblasts in tibial subchondral bone. Data are shown as mean ± s.d. and analyzed by Student’s *t* test. *n* ≥ 6, **P < 0.01

The Electronic supplementary material has been updated to correct Supplementary Fig. 3g.

The original article^1^ was updated.
