# Supplementary material for: Activation of mTORC1 in subchondral bone preosteoblasts promotes osteoarthritis by stimulating bone sclerosis and secretion of CXCL12
Source: Bone Res. 2019 Feb 20;7:5. doi: 10.1038/s41413-018-0041-8 (PMC6381187; doi:10.1038/s41413-018-0041-8)
Supplement: Supplementary file 1 — Supplemental Figure legends and Figure [file 41413_2018_41_MOESM1_ESM.docx]

Activation of mTORC1 in subchondral bone preosteoblasts promotes osteoarthritis by stimulating bone sclerosis and secretion of CXCL12

Chuangxin Lin^1,3#^, Liangliang Liu^1#^, Chun Zeng^1#^, Zhong-Kai Cui^2^, Yuhui Chen^1^, Pinling Lai^1,2^, Hong Wang^1^, Yan Shao^1^, Haiyan Zhang^1^, Rongkai Zhang^1^, Chang Zhao^1^, Hang Fang^1^, Daozhang Cai^1^*, Xiaochun Bai^1,2^*

^1^Department of Orthopedics, Academy of Orthopedics- Guangdong Province, The Third Affiliated Hospital of Southern Medical University, Guangzhou 510630, China;

^2^Key Laboratory of Mental Health of the Ministry of Education, Department of Cell Biology, School of Basic Medical Sciences, Southern Medical University, Guangzhou 510515, China;

^3^Department of Orthopedic Surgery, Shantou Central Hospital, Affiliated Shantou Hospital of Sun Yat-Sen University, Shantou 515041, China.

^#^Chuangxin Lin, Liangliang Liu and Chun Zeng contributed equally to this work.

***Correspondence**: Prof. Daozhang Cai, Department of Orthopaedics, The Third Affiliated Hospital of Southern Medical University, Guangzhou 510630, China. (Phone: +86-20-62784303, Fax: +86-20-62784308, Email: [cdz@smu.edu.cn);](mailto:cdz@smu.edu.cn);) Prof. Xiaochun Bai , Academy of Orthopaedics, Guangdong Province, The Third Affiliated Hospital of Southern Medical University, Guangzhou 510630, China.( Phone: +86-20-61648724, Fax: +86-20-61648208, Email: [baixc15@smu.edu.cn](mailto:baixc15@smu.edu.cn)).


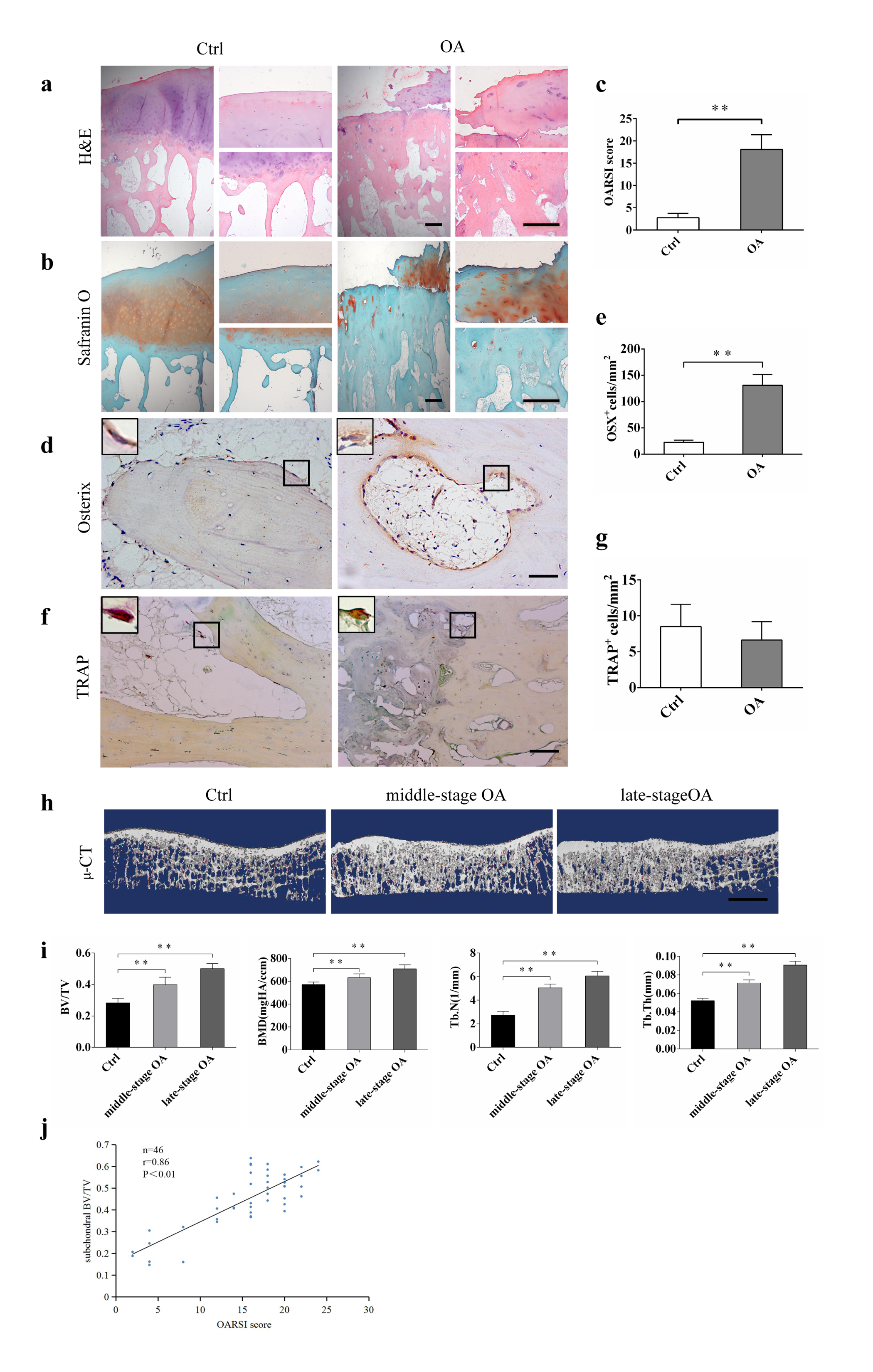


**Supplemental Figure 1. Aberrant bone formation is increased in human tibia subchondral bone during OA progression.** (a) Representative H&E and (b) Safranin O-Fast green staining of [coronal](C:/Users/hp/AppData/Local/youdao/dict/Application/7.5.0.0/resultui/dict/?keyword=coronal) sections of tibia articular cartilage and subchondral bone specimens from OA patients compared to that of non-OA patients (Ctrl). Scale bars, 100 μm. (c) OARSI scores based on the histology analysis of specimens from OA patients compared to that of non-OA patients. (d-g) Representative images and quantitative analysis of immunostaining of Osterix^+^ cells and TRAP staining of osteoclasts in tibial subchondral bone of OA patients and controls. Boxed area is magnified on the top corner. Scale bars: 50 μm (d), 100 μm (f). (h) Representative 3D reconstructed micro-CT images of [coronal](C:/Users/hp/AppData/Local/youdao/dict/Application/7.5.0.0/resultui/dict/?keyword=coronal) sections of subchondral bone medial compartment of OA patients compared to that of non-OA patients. Scale bars, 1 cm. (i) Quantitative analysis of structural parameters of subchondral bone from CT analysis: bone volume/tissue volume (BV/TV), bone mineral density (BMD), trabecular number (Tb.N) and trabecular thickness (Tb.Th). (j) Tibia subchondral BV/TV was positively correlated with OARSI scores analyzed by Spearman’s correlation coefficient test (r = 0.863，*P* < 0.001). Data are shown as mean ± s.d. and analyzed by Student’s t test or one-way ANOVA . n ≥ 8, ***P < 0.01.*


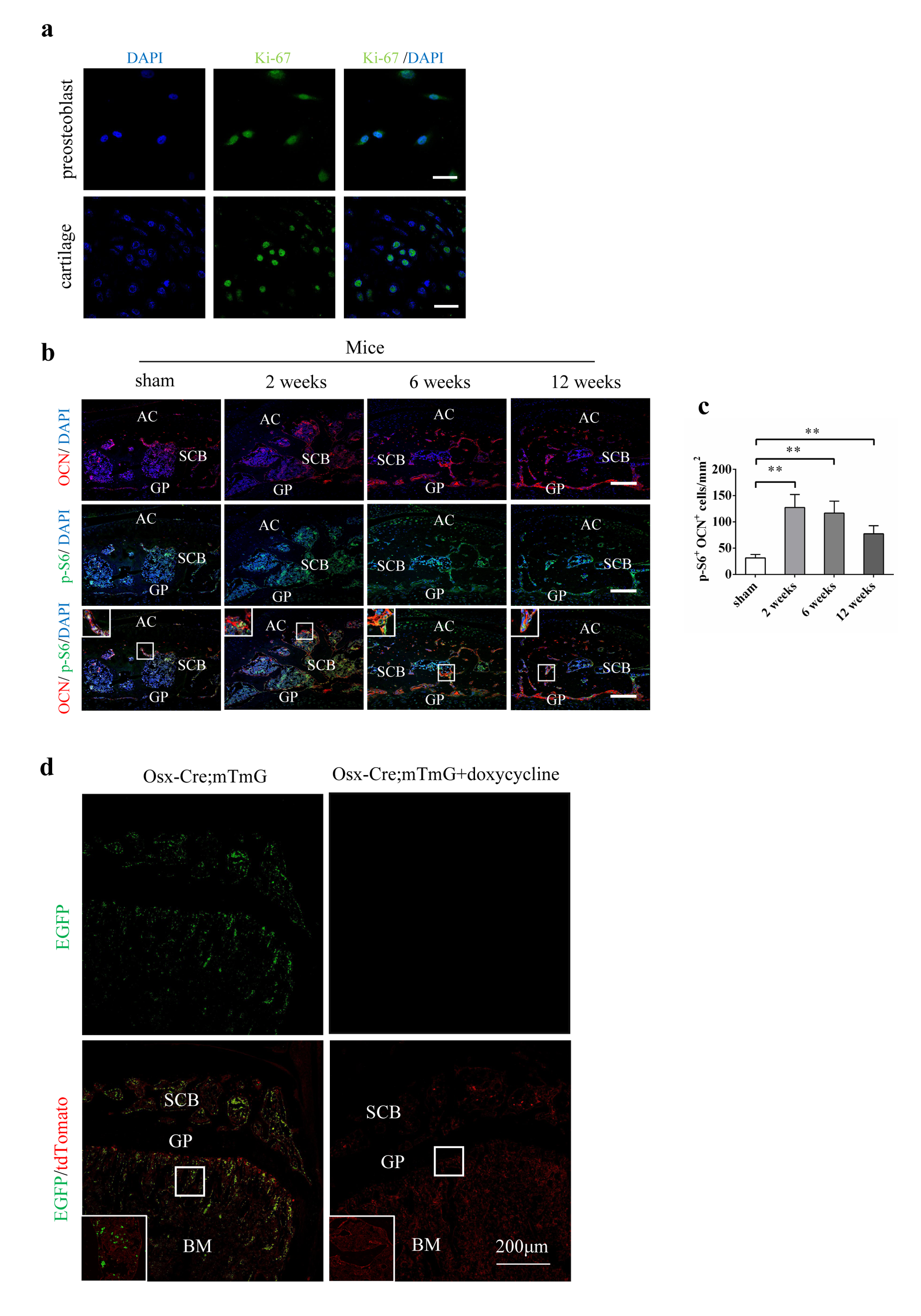


**Supplemental Figure 2. mTORC1 is activated in subchondral bone preosteoblasts in OA development.** (a) Immunofluorescent staining of Ki-67 in cultured primary preosteoblasts and mouse articular cartilage. Scale bars, 20 μm. (b-c) Representative immunofluorescent and quantitative analysis of p-S6 in OCN^+^ osteoblasts in tibial subchondral bone OA mice. Boxed area is magnified on the top corner. Scale bars, 100 μm. AC, articular cartilage; SCB, subchondral bone; GP, growth plate. (d) Osx-Cre targets osteoblast lineage cells. Confocal fluorescent images of EGFP and EGFP/tdTomato on longitudinal tibial sections from three-month-old Osx-Cre;R26-mT/mG mice administrated with or without doxycycline. SCB, subchondral bone; GP, growth plate; BM, bone marrow. Scale bars, 200 μm. Data are shown as mean ± s.d. and analyzed by one-way ANOVA . n ≥ 8, ***P < 0.01*.

**Supplemental Figure 3. mTORC1 activation in preosteoblasts promotes aberrant subchondral bone formation and OA development in mice.** (a) Generation and phenotypes of Osx1-GFP::Cre^TG/+^ TSC1^flox/flox^ mice. PCR analysis confirmed the presence of the Osx1-GFP::Cre^TG/+^ transgene in tissues from homozygote TSC1^flox/flox^ mice (ΔTSC1). (b) 12-week-old ΔTSC1 mice and their TSC1^flox/flox^ littermates. (c) Western blot analysis of p-S6 expression in tibia subchondral bone tissues from ΔTSC1 mice after TSC1 deletion and their TSC1^flox/flox^ littermates (Ctrl). (d) Representative H&E and Safranin O-Fast green staining of sagittal sections of knee joint of 12-weeks-old ΔTSC1 mice after TSC1 deletion vs. their littermates (Ctrl). Scale bars,100μm. Representative micro-CT images of sagittal and coronal views of tibia subchondral bone. Scale bars, 1mm. (e-f) Quantitative analysis of bone mass in subchondral bone: bone volume / total volume (BV/TV) and bone mineral density (BMD). (g-i) Immunostaining of Osterix^+^ cells and TRAP staining of osteoclasts in tibial subchondral bone of 12-weeks-old ΔTSC1 mice after TSC1 deletion vs. their littermates (Ctrl). Quantitative analysis of Osterix^+^ cells or TRAP^+^ cells per square millimeter (mm^2^). Scale bars, 100 μm. Data are shown as mean ± s.d. and analyzed by Student’s t test. n ≥ 6, ***P < 0.01.*


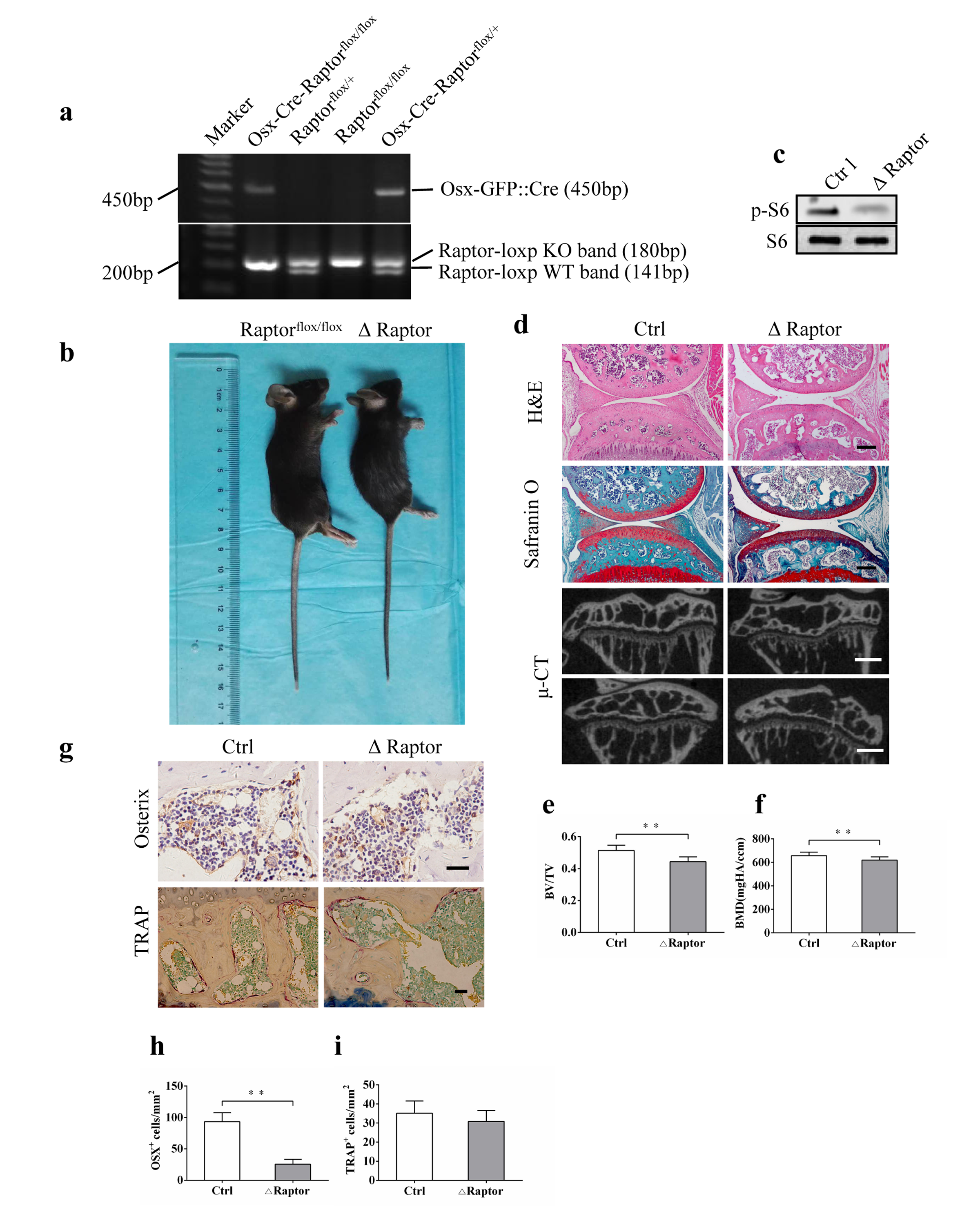


**Supplemental Figure 4. Down-regulated mTORC1 activation in preosteoblasts prevents aberrant subchondral bone formation and OA development in mice.** (a) Generation and phenotypes of Osx1-GFP::Cre^TG/+^ Raptor^flox/flox^ mice. PCR analysis confirmed the presence of the Osx1-GFP::Cre^TG/+^ transgene in tissues from homozygote Raptor^flox/flox^ mice (ΔRaptor). (b) 12-week-old ΔRaptor mice and their Raptor^flox/flox^ littermates. (c) Western blot analysis of p-S6 expression in tibia subchondral bone tissues from ΔRaptor mice after Raptor deletion and their littermates (Ctrl). (d) Representative H&E and Safranin O-Fast green staining of sagittal sections of knee joint of 12-weeks-old ΔRaptor mice after Raptor deletion vs. their littermates (Ctrl). Scale bars,100μm. Representative micro-CT images of sagittal and coronal views of tibia subchondral bone. Scale bars, 1mm. (e-f) Quantitative analysis of bone mass in subchondral bone: bone volume / total volume (BV/TV) and bone mineral density (BMD). (g-i) Immunostaining of Osterix^+^ cells and TRAP staining of osteoclasts in tibial subchondral bone of 12-weeks-old ΔRaptor mice after Raptor deletion vs. their littermates (Ctrl). Quantitative analysis of Osterix^+^ cells or TRAP^+^ cells per square millimeter (mm^2^). Scale bars, 100 μm. Data are shown as mean ± s.d. and analyzed by Student’s t test. n ≥ 6, ***P < 0.01.*
